# Sex differences in the network structures of depressive symptom profiles in Asian patients with depressive disorders: findings from the Research on Asian Psychotropic Patterns for Antidepressants, Phase 3

**DOI:** 10.1017/neu.2025.10020

**Published:** 2025-06-20

**Authors:** Han Seul Kim, Seonjae Lee, Jeongha Lee, Tae Young Choi, Sung-Won Jung, Hyung-Jun Yoon, Hyun Soo Kim, Yangsik Kim, Hyun-Ju Yang, Narae Jeong, Eunsoo Moon, Daeho Kim, Tian-Mei Si, Roy Abraham Kallivayalil, Andi J. Tanra, Amir Hossein Jalali Nadoushan, Kok Yoon Chee, Afzal Javed, Kang Sim, Pornjira Pariwatcharakul, Mian-Yoon Chong, Toshiya Inada, Shih-Ku Lin, Norman Sartorius, Naotaka Shinfuku, Takahiro A. Kato, Jae-Hon Lee, Seon-Cheol Park

**Affiliations:** 1 Department of Psychiatry, Hanyang University Medical Centre, Seoul, Republic of Korea; 2 Department of Psychiatry, Catholic University of Daegu School of Medicine, Daegu, Republic of Korea; 3 Department of Psychiatry, Keimyung University Dongsan Hospital, Daegu, Republic of Korea; 4 Department of Psychiatry, Chosun University Hospital, Gwangju, Republic of Korea; 5 Department of Psychiatry, College of Medicine, Dong-A University, Busan, Republic of Korea; 6 Department of Psychiatry, Inha University Hospital, College of Medicine, Inha University, Incheon, Republic of Korea; 7 Department of Psychiatry, Jeju National University College of Medicine, Jeju, Republic of Korea; 8 Department of Psychology, College of Social Sciences, Daegu University, Gyeongsan, Republic of Korea; 9 Department of Psychiatry, Pusan National University School of Medicine, Yangsan, Republic of Korea; 10 Department of Psychiatry, Hanyang University Guri Hospital, Guri, Republic of Korea; 11 Department of Premedicine, Hanyang University College of Medicine, Seoul, Republic of Korea; 12 Peking University Sixth Hospital, Peking University Institute of Mental Health, NHC Key Laboratory of Mental Health (Peking University), National Clinical Research Centre for Mental Disorders (Peking University Sixth Hospital), Beijing, China; 13 Pushpagiri Institute of Medical Sciences and Research Centre, Thiruvalla and Mar Sleeva Medicity, Palai, Kerala, India; 14 Department of Psychiatry, Faculty of Medicine, Hasanuddin University, Makassar, Indonesia; 15 Mental Health Research Centre, Psychosocial Health Research Institute, Department of Psychiatry, School of Medicine, Iran University of Medical Sciences, Tehran, Iran; 16 Department of Psychiatry & Mental Health, Tunku Abdul Rahman Institute of Neurosciences, Kuala Lumpur Hospital, Kuala Lumpur, Malaysia; 17 Pakistan Psychiatric Research Centre, Fountain House, Lahore, Pakistan; 18 Buangkok Green Medical Park, Institute of Mental Health, Singapore, Singapore; 19 Department of Psychiatry, Faculty of Medicine Siriraj Hospital, Mahidol University, Bangkok, Thailand; 20 Regency Specialist Hospital, Johor, Malaysia; 21 Department of Psychiatry, Nagoya University Graduate School of Medicine, Nagoya, Aichi, Japan; 22 Department of Psychiatry, Linkou Chang Gung Memorial Hospital, Taoyuan, Taiwan; 23 Taipei City Hospital and Psychiatric Centre, Taipei, Taiwan; 24 Association for the Improvement of Mental Health Programs, Geneva, Switzerland; 25 School of Human Sciences, Seinan Gakuin University, Fukuoka, Japan; 26 Department of Psychiatry, Hokkaido University Graduate School of Medicine, Sapporo, Japan; 27 Department of Psychiatry, Schulich School of Medicine and Dentistry, Western University, London, ON, Canada; 28 Hanyang Institute of Bioscience and Biotechnology, Hanyang University, Seoul, Republic of Korea

**Keywords:** Depressive disorders, depressive symptoms, sex differences, Asian population, network analysis

## Abstract

**Background:**

Depression is a complex mental health disorder with highly heterogeneous symptoms that vary significantly across individuals, influenced by various factors, including sex and regional contexts. Network analysis is an analytical method that provides a robust framework for evaluating the heterogeneity of depressive symptoms and identifying their potential clinical implications.

**Objective:**

To investigate sex-specific differences in the network structures of depressive symptoms in Asian patients diagnosed with depressive disorders, using data from the Research on Asian Psychotropic Prescription Patterns for Antidepressants, Phase 3, which was conducted in 2023.

**Methods:**

A network analysis of 10 depressive symptoms defined according to the National Institute for Health and Care Excellence guidelines was performed. The sex-specific differences in the network structures of the depressive symptoms were examined using the Network Comparison Test. Subgroup analysis of the sex-specific differences in the network structures was performed according to geographical region classifications, including East Asia, Southeast Asia, and South or West Asia.

**Results:**

A total of 998 men and 1,915 women with depression were analysed in this study. The analyses showed that all 10 depressive symptoms were grouped into a single cluster. Low self-confidence and loss of interest emerged as the most central nodes for men and women, respectively. In addition, a significant difference in global strength invariance was observed between the networks. In the regional subgroup analysis, only East Asian men showed two distinct clustering patterns. In addition, significant differences in global strength and network structure were observed only between East Asian men and women.

**Conclusion:**

The study highlights the sex-specific differences in depressive symptom networks across Asian countries. The results revealed that low self-confidence and loss of interest are the main symptoms of depression in Asian men and women, respectively. The network connections were more localised in men, whereas women showed a more diverse network. Among the Asian subgroups analysed, only East Asians exhibited significant differences in network structure. The considerable effects of neurovegetative symptoms in men may indicate potential neurobiological underpinnings of depression in the East Asian population.

Significant outcomes

- Network analysis revealed sex-specific differences in the centrality of depressive symptoms in Asian patients with depressive disorders. Self-blame was the most central in men, while loss of interest held the highest centrality in women.
- Since, the overall network structure appeared similar across sexes, depressive symptoms in both group formed a single, densely interconnected cluster. They illustrate the complexity of their interrelations.
- By applying network analysis to a large, multi-national sample of Asian patients, the study provides novel insights into sex-based differences in depressive symptom profiles across diverse cultural contexts.

Limitations

- The cross-sectional design of the study restricts the ability to draw causal conclusions about the relationships among depressive symptoms.
- The use of self-reported measures may introduce response biases, potentially compromising the accuracy of symptom interrelations.
- Since, potential confounding factors have not been not controlled, they may have contributed to the sex-specific differences observed in the depressive symptom network.

## Introduction

Depression involves a broad range of symptoms spread across affective, cognitive, and neurovegetative domains, reflecting its heterogeneity and complex nature (Park & Kim 2019a). Due to the inherent heterogeneity of depressive symptoms and their neurobiological underpinnings, approximately 30% of patients fail to achieve remission even after multiple advanced pharmacological interventions are applied (Sackeim, 2001). The heterogeneity of depression has been attributed to various factors, including genetic, epigenetic, neurobiological, and environmental factors. Among these factors, sex plays a critical role in shaping the symptom presentation, progression, and treatment outcomes of depression (Lynch *et al*., 2020). Previous studies have demonstrated how depressive symptoms and patterns vary between men and women (Piccinelli & Wilkinson, 2000). Women often report mild to moderate depressive symptoms along with more typical depressive symptoms, such as depressive mood, appetite disturbances/weight changes, and sleep disturbances. In contrast, men are more likely to exhibit severe depression and poor impulse control, engaging in risk-taking behaviours such as substance abuse, which may obscure traditional depressive symptoms (Cavanagh *et al*., 2017; Shi *et al*., 2021). This may be related to differences in the structural and functional organisation of the brain between men and women. Recent neuroimaging studies have shown that women tend to exhibit greater activity in areas associated with emotional regulation, whereas men show strong activation in regions related to stress responses and aggression. These neurobiological differences may help explain the sex-specific differences in depression symptomatology. For example, women are more prone to internalising symptoms, such as sadness, whereas men mostly show externalised behaviours, such as anger or substance abuse (Borsboom *et al*., 2019; Mohammadi *et al*., 2023). Regarding geographical or environmental factors, several studies have been conducted to explore the genetic heterogeneity of depression across different regions. One such study identified novel depression-related genes in East Asian populations. The study demonstrated that only 11% of the depression-associated genes previously identified in European populations were significantly replicated in East Asians (Giannakopoulou *et al*., 2023).

The complexity of depressive symptoms has led to the adoption of network analysis, which involves viewing psychological attributes not as outcomes of a single latent cause but as components within a complex system of dynamic interactions among symptoms (Guyon *et al*., 2017; Saxe, 2017; Contreras *et al*., 2019; Lee *et al*., 2021). Network analysis can identify ‘high centrality symptoms’, highlighting clinical implications that allow for the selection of interventions focused on these key symptoms, thereby facilitating the alleviation of associated symptoms and improvement of therapeutic outcomes ( McNally 2016; Saxe *et al*., 2016; Borsboom *et al*., 2019). Sex-specific differences in depressive symptoms have been analysed in various studies conducted using network analysis. These studies had diverse sample sizes and recruitment methods, ranging from hundreds of clinical participants to non-clinical populations exceeding 200,000 participants. The findings of these studies suggest that somatic symptoms (e.g., fatigue, concentration difficulties) and suicidal ideation are central among men. In contrast, symptoms such as guilt and hopelessness are consistently central among women, with stronger associations between depressive symptoms and anxiety. The overall structure of depressive networks in men and women is similar across studies. However, these studies demonstrated that women show greater network stability and more densely connected and stronger networks, particularly around emotional symptoms, whereas men exhibit networks mostly focused on somatic and self-efficacy-related symptoms (Vetter *et al*., 2021; Jin *et al*., 2022; Alcalde *et al*., 2024).

The Research on Asian Psychotropic Prescription Patterns for Antidepressants (REAP-AD) is a study with a large sample size conducted to investigate the clinical factors that affect antidepressant prescription patterns within an Asian regional context. Previous REAP-based studies have highlighted differences in depressive symptoms according to sex and region. A study on sex-specific differences in depressive symptoms, conducted without using network analysis, revealed that men are more likely to experience loss of interest and fatigue, whereas women are more prone to report suicidal thoughts or actions. However, the study showed no notable sex-specific differences in other depressive symptoms, such as persistent sadness, poor concentration, and insomnia (Park *et al*., 2015; Kim & Park, 2021). In contrast, a study that focused on regional differences in depressive symptoms revealed that sadness, fatigue, and interest loss are the top three central symptoms of depression within the general population. However, subtle variations were observed when the study population was analysed according to East, South, and Southeast Asian regions. For the East Asians, the top three central symptoms within the network were observed to be guilt, fatigue, and suicidal ideation. For the South and Southeast Asians, the central symptoms were sadness, sleep disturbances, and loss of interest. For Southeast Asians, the primary symptoms included sadness, self-esteem issues, and concentration difficulties (Borsboom *et al*., 2019). Considering the findings of these previous studies, we conducted the current study to evaluate the sex-specific differences in the network structure and centrality of depressive symptoms using network analysis. In addition, we sought to examine the influence of regional variations on the symptom networks by conducting subgroup analyses according to different regions in Asia. This study was conducted using a large, multinational sample of Asian patients with depressive disorders while identifying the similarities and differences in differences in findings compared to previous studies.

## Methods

### Study design and participants

The REAP-AD study is a large-scale, multinational, collaborative study conducted between August 2023 and February 2024. A total of 4,587 patients with psychiatric disorders recruited from various regions were included in the study (Park *et al*., 2015). The regions in Asia were classified based on United Nations classification as follows: East Asia (China, Hong Kong, Japan, South Korea, and Taiwan), Southeast Asia (Indonesia, Malaysia, Singapore, and Thailand), and South or West Asia (India, Iran, and Pakistan). The study was coordinated by the REAP consortium, and data collection was managed through a secure web-based platform hosted by Taipei City Hospital. The study protocols and consent forms were approved by the Institutional Review Boards of the survey centres, including Hanyang University Guri Hospital, Guri, Republic of Korea (receipt number: 2023-05-021-006). All memoranda of understanding were signed before the commencement of the study. All the participating countries shared de-identified, anonymised data via a secure web-based platform hosted by the coordinating site and secretariat at Taipei City Hospital.

Participants were selected based on their use of antidepressant medication during the survey period. The specific inclusion criteria were as follows: (i) age between 10 and 80 years, (ii) a confirmed diagnosis of depressive disorders by a clinical psychiatrist based on the International Classification of Diseases, Tenth Revision (ICD-10) (World Health Organization, 1992), and (iii) use of at least one antidepressant, classified according to the anatomical therapeutic chemical system developed by the WHO Collaborating Centre for Drug Statistics Methodology (World Health Organization, 2018). Inpatients and outpatients were included to ensure that the study population was diverse. Informed consent was obtained from all the participants. The exclusion criteria were as follows: (i) having a medical or neurological condition severe enough to hinder the accuracy of study assessments or interviews, (ii) age<10 years or>80 years, and (iii) illiteracy or inability to provide informed consent. Clinical psychiatrists collected demographic, clinical, and prescription data at each survey centre. Diagnoses were made according to the ICD-10, and depressive disorders were classified as follows: F32 (Depressive Episode), F33 (Recurrent Depressive Disorder), or F34 (Persistent Mood Disorders) (Kessler & Üstün, 2004). Comorbid psychiatric disorders were determined based on the ICD-10. A total of 2,913 participants (1,915 women and 998 men) diagnosed with depression were included in this study.

### Depressive symptom profiles and other psychometric assessment scales

This study was conducted using 10 depressive symptom profiles outlined in the National Institute for Health and Care Excellence (NICE) guidelines, which emphasise practical and holistic approaches for clinical application. The symptoms were assessed by clinical psychiatrists and included persistent sadness or low mood (SAD), loss of interest or pleasure (INT), fatigue or low energy (ENE), disturbed sleep (SLE), poor concentration or indecisiveness (CON), low self-confidence (SEF), poor or increased appetite (APP), suicidal thoughts or acts (SUI), agitation or slowing of movements (AGI), and guilt or self-blame (GUI) (National Institute for Health and Care Excellence, 2022).

Depressive symptoms and anxiety levels among the study participants were evaluated using the Patient Health Questionnaire-9 (PHQ-9) and Generalised Anxiety Disorder-7 (GAD-7), respectively (Spitzer *et al*., 1999; Sapra *et al*., 2020). The validity and reliability of the PHQ-9 and GAD-7 have been systematically examined, and standardised versions of these tools in various Asian languages have been developed (Lotrakul *et al*., 2008; Poongothai *et al*., 2009; Park *et al*., 2010; Sherina *et al*., 2012; Zeng *et al*., 2013; Wang *et al*., 2014; Ahmad *et al*., 2017; Ahmad *et al*., 2018; Dadfar *et al*., 2018; Doi *et al*., 2018; Muramatsu *et al*., 2018; De Man *et al*., 2021; Lee *et al*., 2022; Pheh *et al*., 2023).

### Statistical analysis

The R-package qgraph was used to analyse sex-specific differences in the network structures of depressive symptoms (Epskamp *et al*., 2012; Burger *et al*., 2023). Network structures, consisting of nodes (representing depressive symptoms) and edges (representing associations among nodes), were constructed using the data subsets for men and women from the 2023 study year. The Least Absolute Shrinkage and Selection Operator (LASSO) was used to control for false-positive edges, with very small edges set to zero (Tibshirani, 1996).

As the graphical LASSO (GLASSO) procedure was used in this study, edges represented partial correlation coefficients, meaning that the average edge represents the relationship level between two symptoms while controlling for all other relationships within the network. The shrinkage parameter was minimised using the Extended Bayesian Information Criterion, ensuring accurate recovery of the underlying network structures (Chen & Chen, 2008; Van Borkulo *et al*., 2014). The Fruchterman–Reingold algorithm visualised the network structure, ensuring that closely related nodes were positioned together.

All NICE guideline items were considered ordered categorical variables, and the network estimation was based on polychoric correlations. A community-detecting algorithm was applied to assess whether the number and weighted strength of the edges within a cluster exceeded those in other clusters, reflecting community structures in the network (Reichardt & Bornholdt, 2006). The spin-glass community detection function in the R-package igraph was used to evaluate the GLASSO network (parameters: weights = null, vertex = null, parupdate = false, gamma = 0.5, start temperature = 1, stop temperature = 0.01, cooling factor = 0.99, spins = 17) (Reichardt & Bornholdt, 2006). The centrality of each node was determined using multiple metrics. Node strength centrality, defined as the sum of all associations between a given node and all other nodes (i.e., the sum of all edge weights connected to a node), is regarded as a common and stable centrality metric and emphasised as the primary measure. Closeness centrality indicates how close one symptom is to all other symptoms, reflecting the overall integration of a symptom within the network. Betweenness centrality measures the number of shortest paths connecting two other nodes that pass through the node under consideration, highlighting nodes that act as bridges within the network (Epskamp *et al*., 2018).

The stability of each network was assessed using bootstrapping methods, with 1,000 bootstraps performed to calculate correlation stability (CS) coefficients. The CS coefficient denotes the maximum proportion of cases that can be eliminated while maintaining a 95% probability that the correlation between the centrality indices of the original and subset networks will remain above 0.700, indicating stable and reliable centrality estimates (Cohen, 2013). A CS coefficient above 0.250, preferably above 0.500, ensures the robustness of the results (Epskamp *et al*., 2018). Moreover, 95% nonparametric bootstrap confidence intervals were used to compare centrality indices and determine significant differences.

The Network Comparison Test (NCT) with 1,000 iterations was used for robust results to reveal sex-specific differences in the network structures of depressive symptoms in men and women using the R package NCT (Cohen, 2013). The networks were compared in terms of network structure invariance and global strength invariance. Spearman’s correlation coefficient was used to assess the correlation of node centrality strength and edge weights between the two networks. *P*-value <0.05 indicated a robust and significant difference (Cohen, 2013). Further subgroup analyses were conducted to compare sex-specific differences in depressive symptom network structures across different regions of Asia, classified into three categories: East Asia, Southeast Asia, and South or West Asia. Each subgroup was analysed using separate NCTs with 1,000 iterations, following the same analytical procedures described above. All statistical analyses were conducted using R 4.3.3 (R Foundation for Statistical Computing, Vienna, Austria).

## Results

### General characteristics of the study participants

The general and baseline clinical characteristics of the 2,913 participants are summarised in Table 1. Men and women with depressive disorders accounted for 34.3% (*n* = 998) and 65.7% (*n* = 1,915) of the study population, respectively. The mean age of the participants was 39.3 years (SD = 16.7). Regarding the participants’ countries, Pakistan had the highest number of participants (15.7%, *n* = 458), followed by China (13.6%, *n* = 396), Indonesia (12.3%, *n* = 357), and Malaysia (11.1%, *n* = 324). Most participants were outpatients (81.7%, *n* = 2,380); only 18.0% (*n* = 523) were inpatients. Regarding the severity of depressive symptoms, moderate and severe symptoms were observed in 27.5% (*n* = 800) and 28.1% (n = 819) of the participants, respectively. Men showed higher rates of subthreshold and mild symptom severity (46.7% vs. 43.2%), whereas women exhibited higher rates of moderate and severe symptom severity (56.8% vs. 53.4%). Anxiolytics were the most frequently prescribed medications (37.5%, *n* = 1,093), followed by antipsychotics (32.0%, *n* = 931), hypnotics (16.6%, *n* = 484), and mood stabilisers (6.2%, *n* = 182).

**Table 1.** Baseline and clinical characteristics of the participants

	All participants (*n* = 2,913)	Men (*n* = 998)	Women (*n* = 1,915)
Age, mean (SD) years	39.3 (16.7)	38.8 (16.0)	39.5 (17.0)
Country, *n* (%)			
China	396 (13.6)	165 (16.5)	231 (12.1)
India	178 (6.1)	64 (6.4)	114 (6.0)
Indonesia	357 (12.3)	132 (13.2)	225 (11.7)
Iran	157 (5.4)	47 (4.7)	110 (5.7)
Japan	220 (7.6)	84 (8.4)	136 (7.1)
Malaysia	324 (11.1)	106 (10.6)	218 (11.4)
Pakistan	458 (15.7)	139 (13.9)	319 (16.7)
Singapore	44 (1.5)	11 (1.1)	33 (1.7)
South Korea	201 (6.9)	51 (5.1)	150 (7.8)
Taiwan	256 (8.8)	93 (9.3)	163 (8.5)
Thailand	322 (11.1)	106 (10.6)	216 (11.3)
Regional subgroups, *n* (%)			
East Asia	1,073 (36.8)	393 (39.4)	680 (35.5)
Southeast Asia	1,047 (35.9)	355 (35.6)	692 (36.1)
South and West Asia	793 (27.2)	250 (25.1)	543 (28.4)
Treatment setting, *n* (%)			
Inpatient	523 (18.0)	195 (19.5)	328 (17.1)
Outpatient	2,380 (81.7)	798 (80.0)	1,582 (82.6)
Severity of depressive symptoms, *n* (%)			
Subthreshold	869 (29.8)	311 (31.2)	558 (29.1)
Mild	425 (14.6)	155 (15.5)	270 (14.1)
Moderate	800 (27.5)	256 (25.7)	544 (28.4)
Severe	819 (28.1)	276 (27.7)	543 (28.4)
PHQ-9 score, *n* (%)			
Minimal	863 (29.6)	313 (31.4)	550 (28.7)
Mild	548 (18.8)	205 (20.5)	343 (17.9)
Moderate	1,071 (36.8)	354 (35.5)	717 (37.4)
Severe	431 (14.8)	126 (12.6)	305 (15.9)
GAD-7 score, *n* (%)			
Minimal	1,159 (39.8)	427 (42.8)	732 (38.2)
Mild	697 (23.9)	259 (26.0)	438 (22.9)
Moderate	558 (19.2)	176 (17.6)	382 (19.9)
Severe	499 (17.1)	136 (13.6)	363 (19.0)
Use of psychotropic drugs			
Antipsychotic, *n* (%)	931 (32.0)	335 (33.6)	596 (31.1)
Mood stabiliser, *n* (%)	182 (6.2)	74 (7.4)	108 (5.6)
Anxiolytic, *n* (%)	1,093 (37.5)	385 (38.6)	708 (37.0)
Hypnotic, *n* (%)	484 (16.6)	168 (16.8)	316 (16.5)

GAD-7, Generalised Anxiety Disorder-7; PHQ-9, Patient Health Questionnarie-9.

The distribution of the participants who experienced each of the 10 depressive symptoms, defined based on the NICE guidelines, is summarised in Table 2. SAD was the most prevalent symptom in both groups, affecting 85.8% of the women and 82.2% of the men, followed by INT (68.5% of the women and 69.3% of the men) and ENE (60.3% of the women and 60.5% of the men). Conversely, AGI was the least prevalent symptom in both groups (28.1% of the women and 27.8% of the men), followed by GUI (30.4% of the women and 29.1% of the men).

**Table 2.** Response frequency rates for the depressive symptom profiles defined according to the NICE guidelines

	All participants (*n* = 2,913)	Men (*n* = 998)	Women (*n* = 1,915)
SAD, *n* (%)	2,463 (84.6)	820 (82.2)	1,643 (85.8)
INT, *n* (%)	2,003 (68.8)	692 (69.3)	1,311 (68.5)
ENE, *n* (%)	1,759 (60.4)	604 (60.5)	1,155 (60.3)
SLE, *n* (%)	1,946 (66.8)	642 (64.3)	1,304 (68.1)
CON, *n* (%)	1,426 (49.0)	503 (50.4)	923 (48.2)
SEF, *n* (%)	1,209 (41.5)	416 (41.7)	793 (41.4)
APP, *n* (%)	1,097 (37.7)	337 (33.8)	760 (39.7)
SUI, *n* (%)	978 (33.6)	283 (28.4)	695 (36.3)
AGI, *n* (%)	816 (28.0)	277 (27.8)	539 (28.1)
GUI, *n* (%)	873 (30.0)	290 (29.1)	583 (30.4)

SAD, persistent sadness or low mood; INT, loss of interest or pleasure; ENE, fatigue or low energy; SLE, disturbed sleep; CON, poor concentration or indecisiveness; SEF, low self-confidence; APP, poor or increased appetite; SUI, suicidal thoughts or acts; AGI, agitation or slowing of movements; GUI, guilt or self-blame.

NICE, National Institute for Health and Care Excellence.

### Estimated network structures of the depressive symptoms analysed according to sex

**Figure 1.**
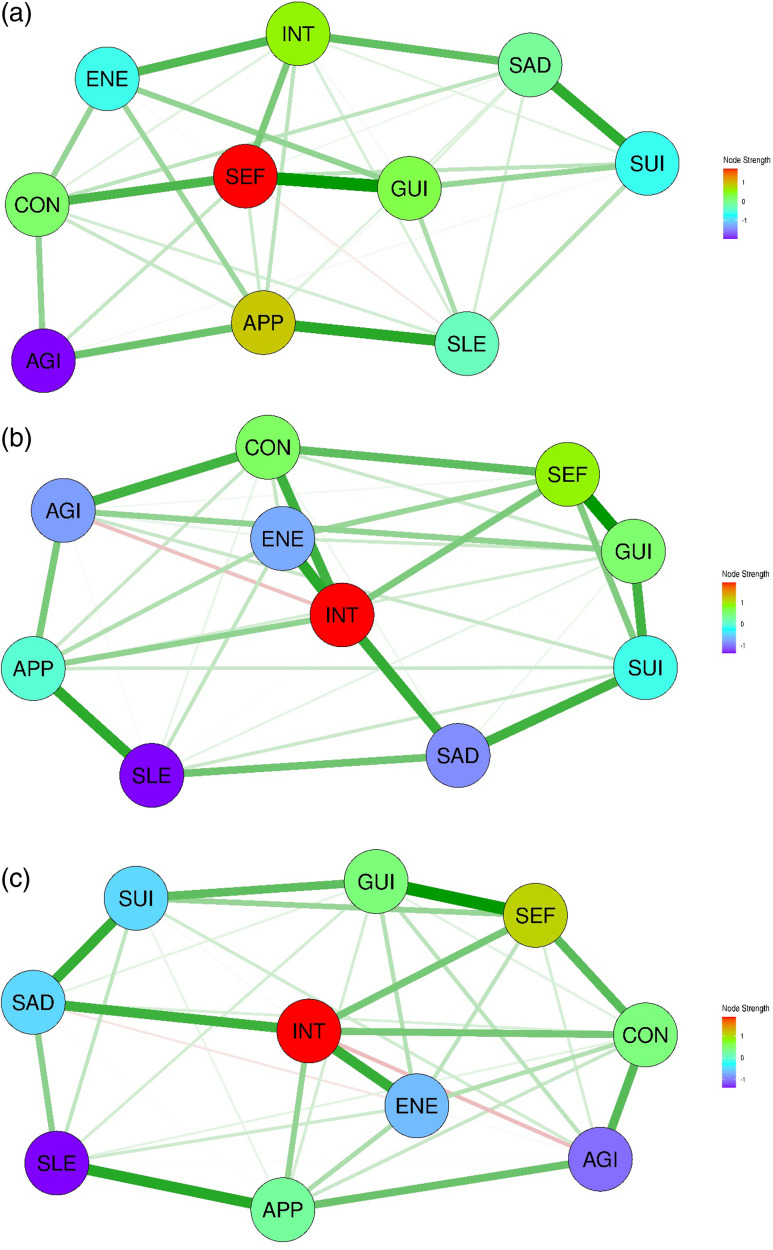
Network structures of 10 depressive symptoms in Asian patients with depression. (a) Network structure of depressive symptoms in Asian men with depression. (b) Network structure of depressive symptoms in Asian women with depression. (c) Network structure of depressive symptoms in Asian patients with depression. SAD, persistent sadness or low mood; INT, loss of interest or pleasure; ENE, fatigue or low energy; SLE, disturbed sleep; CON, poor concentration or indecisiveness; SEF, low self-confidence; APP, poor or increased appetite; SUI, suicidal thoughts or acts; AGI, agitation or slowing of movements; GUI, guilt or self-blam.

Network analysis of both sex-based groups showed that all 10 depressive symptoms are grouped into a single cluster. A total of 36 edges with sufficient stability were identified in the network for men (Fig. 1a). Of the 36 edges, 35 (97.2%) showed positive values, except for the SLE–SEF edge (−0.033). The key interconnections identified included SEF–GUI (weight = 0.356), SLE–APP (0.302), SAD–SUI (0.291), and CON–SEF (0.251). The edge statistics demonstrated a preferred interpretability, with a CS coefficient of 0.517. SEF exhibited the highest node strength centrality, followed by APP, INT, GUI, and CON (Supplementary Figure 1a). In contrast, AGI showed the lowest node strength centrality in the network. Node strength centrality revealed a reasonably interpretable level for the CS coefficient (0.361). The edge strength, with a maximum drop proportion of 0.517, showed moderate stability.

The network structure of the depressive symptoms in women was different from that of men (Fig. 1b). A total of 36 edges with sufficient stability were identified. Of these, 34 (94.4%) showed positive values, except for SAD–ENE (−0.020) and INT–AGI (−0.077). Significant associations among the symptoms, including SEF–GUI (0.310), INT–ENE (0.259), and SLE–APP (0.258), were revealed. The edge statistics demonstrated a preferred interpretability (CS coefficient = 0.672). INT exhibited the highest node strength centrality, followed by SEF, CON, and GUI (Supplementary Figure 1b). In contrast, SLE showed the lowest node strength centrality. Both node strength and closeness centrality exhibited interpretable levels of stability (CS coefficient = 0.284 and 0.439, respectively).

### Comparison of the network structures of the depressive symptoms analysed according to sex

As shown in Table 3, Spearman’s correlation of the network strengths was 0.661, indicating a moderate degree of similarity in node centrality strength between the networks for men and women. The NCT identified significant differences in global strength invariance between the two networks (*p*-value = 0.020). In addition, three edges (6.7%) showed significant differences between the two networks. However, when the sample sizes were equalised, the p-value for global strength invariance was 0.445, indicating no significant difference in network strength between the two networks. Additionally, no significant difference was observed in the network structure invariance test (*p*-value = 0.535). Moreover, the results remained consistent when the sample sizes were equalised (*p*-value = 0.655). Fig. 1(c) shows the network of the depressive symptoms in all the participants with depression. The network revealed a single unified cluster of symptoms. A total of 38 edges with sufficient stability were identified. Of these, 36 (94.7%) showed positive values, except for SAD–ENE (−0.037) and INT–AGI (−0.087), which is consistent with the results observed in the women’s network. The edges that showed significant interconnections included SEF–GUI (0.330), SLE–APP (0.283), INT–ENE (0.262), SAD–SUI (0.261), and SAD–INT (0.252). The network exhibited strong stability, with a CS coefficient of 0.750 for edge strength, 0.517 for node strength, and 0.439 for closeness centrality. INT exhibited the highest node strength centrality, followed by SEF, GUI, CON, and APP (Supplementary Figure 1c). In contrast, SLE showed the lowest node strength centrality.

**Table 3.** Results of pairwise network comparisons of the depressive symptom networks for the entire cohort and the regional subgroups

	Network 1 vs. 2	Network 3 vs. 4	Network 5 vs. 6	Network 7 vs. 8
NCT: network structure invariance (p-value)	0.535	0.011	0.729	0.349
NCT: network structure invariance-equal sample sizes (mean p-value)	0.655	0.140	0.618	0.555
NCT: global strength invariance (p-value)	0.020	0.008	0.562	0.619
NCT: global strength invariance-equal sample sizes (mean p-value)	0.446	0.085	0.584	0.514
No. (% total) of significantly different edges in the individual edge invariance test	3 (6.7)	4 (8.9)	2 (4.4)	1 (2.2)
Spearman correlation: node strength centrality	0.661	−0.091	0.491	0.515
Spearman correlation: edges	0.923	0.742	0.729	0.801
No. (%) of rank-order-difference = 0	20 (44.4)	1 (2.2)	20 (44.4)	1 (2.2)

Network 1, entire Asian cohort (men); Network 2, entire Asian cohort (women); Network 3, East Asia subgroup (men); Network 4, East Asia subgroup (women); Network 5, Southeast Asia subgroup (men); Network 6, Southeast Asia subgroup (women); Network 7, South and West Asia subgroup (men); Network 8, South and West Asia subgroup (women).

### Comparative subgroup analyses of the network structures of the depressive symptoms according to region and sex

#### Sex-specific differences in the network structures of depressive symptoms in East Asians

**Figure 2.**
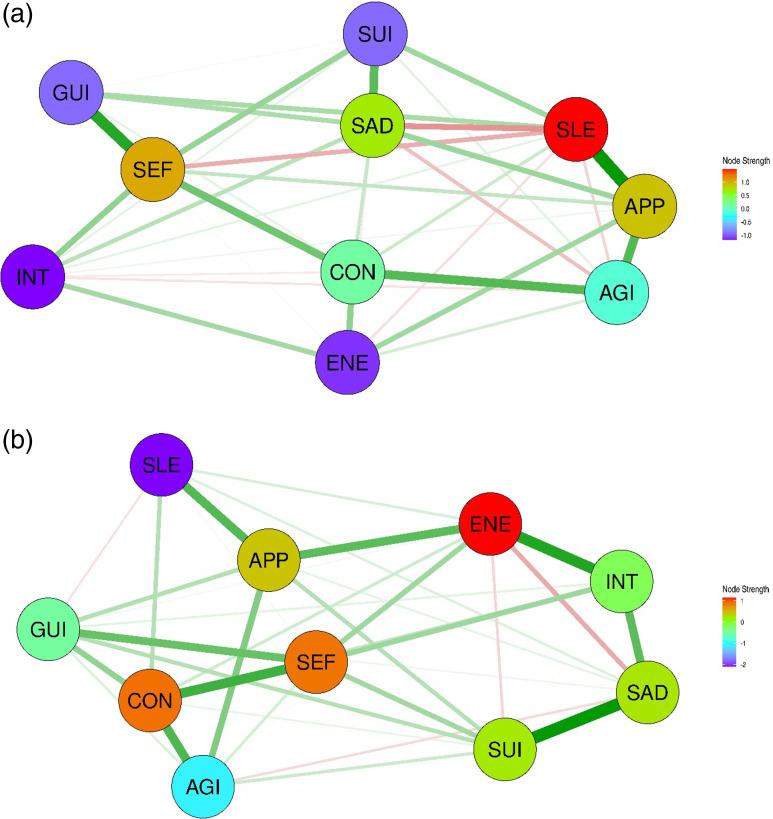
Network structures of 10 depressive symptoms in East Asian patients with depression. (a) Network structure of depressive symptoms in East Asian men with depression. (b) Network structure of depressive symptoms in East Asian women with depression. SAD, persistent sadness or low mood; INT, loss of interests or pleasure; ENE, fatigue or low energy; SLE, disturbed sleep; CON, poor concentration or indecisiveness; SEF, low self-confidence; APP, poor or increased appetite; SUI, suicidal thoughts or acts; AGI, agitation or slowing of movements; GUI, guilt or self-blame.

The baseline and clinical characteristics of East Asian patients with depressive disorders are shown in Supplementary Tables 1 and 2. Network analysis of East Asian men revealed two distinct clustering patterns of depressive symptoms. The first cluster comprised SAD, INT, SEF, SUI, and GUI, whereas the second cluster included ENE, SLE, CON, APP, and AGI. As illustrated in Fig. 2(a), SLE–APP (weight = 0.464), SEF–GUI (0.399), APP–AGI (0.314), CON–AGI (0.312), and SAD–SUI (0.305) showed strong edge weights in the network. The edge statistics demonstrated an interpretable level of stability (CS coefficient = 0.517). However, the node strength showed lower than an interpretable level of stability (CS coefficient = 0.227), whereas SLE and INT showed the highest and lowest node strength centralities, respectively (Supplementary Figure 2a). For East Asian women, all the symptoms form a single cluster in the network. As illustrated in Fig. 2(b), SAD–SUI (weight = 0.402), INT–ENE (0.350), and CON–SEF (0.307) exhibited strong edge weights. Evaluation of the edge strength revealed a high CS coefficient (0.516). Node strength demonstrated moderate stability (CS coefficient = 0.284) (Supplementary Figure 2b). ENE had the highest node strength centrality, followed by CON, SEF, and APP, whereas SLE had the lowest.

The NCT for the East Asian subgroups revealed significant differences in global strength and network structure invariance (*p*-value = 0.008 and *p*-value = 0.011, respectively) (Table 3). When the sample sizes were equalised, the *p*-value for global strength invariance increased to 0.085, which was not statistically significant, even though it suggested a potential difference. The Spearman correlation for the edge weights was relatively high (0.742), and four edges (8.9%) showed significant differences between the two groups. In contrast, the Spearman correlation for node centrality strength was −0.091, and only one node (2.2%) had the same rank order across groups, suggesting a weak association in centrality patterns between the two networks.

#### Sex-specific differences in the network structures of depressive symptoms in Southeast Asia

**Figure 3.**
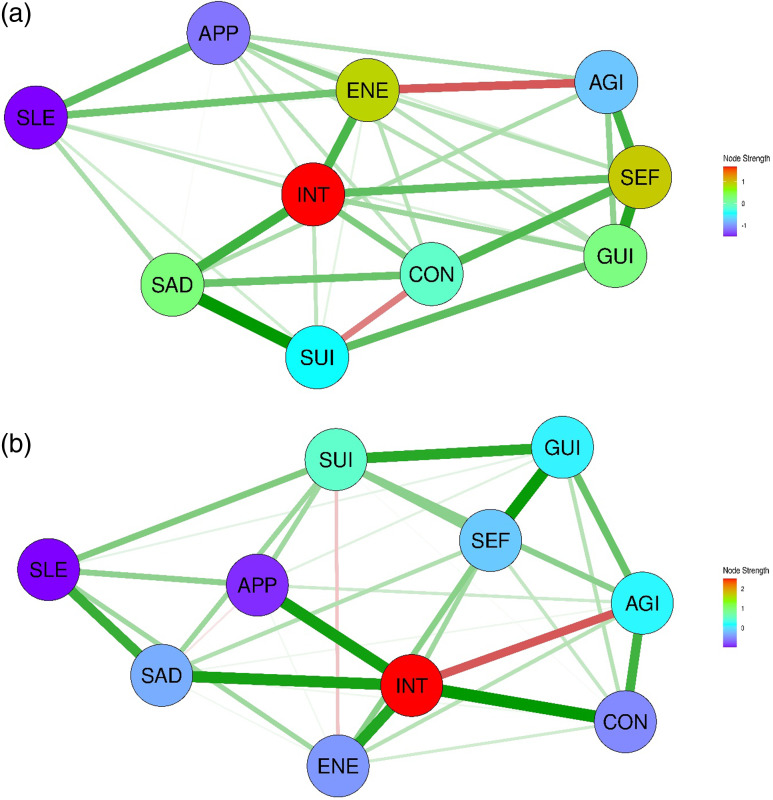
Network structures of 10 depressive symptoms in Southeast Asian patients with depression. (a) Network structure of depressive symptoms in Southeast Asian men with depression. (b) Network structure of depressive symptoms in Southeast Asian women with depression. SAD, persistent sadness or low mood; INT, loss of interest or pleasure; ENE, fatigue or low energy; SLE, disturbed sleep; CON, poor concentration or indecisiveness; SEF, low self-confidence; APP, poor or increased appetite; SUI, suicidal thoughts or acts; AGI, agitation or slowing of movements; GUI, guilt or self-blame.

The baseline and clinical characteristics of East Asian patients with depressive disorders are shown in Supplementary Tables 3 and 4. The networks for both sexes were composed of a single symptom cluster. As shown in Fig. 3(a) and 3(b), the network for the Southeast Asian men showed high edge weights for SAD–SUI (0.335), SEF–GUI (0.288), SAD–INT (0.253), and SEF–AGI (0.252), whereas that for the women showed strong edges for INT–CON (0.328), SEF–GUI (0.319), INT–APP (0.309), and SAD–INT (0.302). INT showed the highest node strength centrality for both sexes, whereas SLE had the lowest node strength centrality, followed by APP (Supplementary Figures 3a and 3b). The CS coefficient for the edge weight in the network for men showed stability of an interpretable level (CS coefficient = 0.285), whereas the network for women indicated strong stability (CS coefficient = 0.517). However, the node strength for men demonstrated stability of lower than stability of an interpretable level (CS coefficient = 0.051), whereas that for women showed stability of an interpretable level (i.e., CS coefficient = 0.361). As shown in Table 3, the results of the NCT indicated no significant differences in either global strength or network structure invariance for the Southeast Asia subgroup, regardless of whether the sample sizes were equalised or not. Moreover, 44.4% of the nodes in the Southeast Asia subgroup shared identical centrality ranks, indicating a high level of concordance. In addition, the Spearman correlation for edge weights was relatively high (0.729), and two edges (4.4%) showed significant differences between the two groups.

#### Sex-specific differences in the network structures of depressive symptoms in South or West Asia

**Figure 4.**
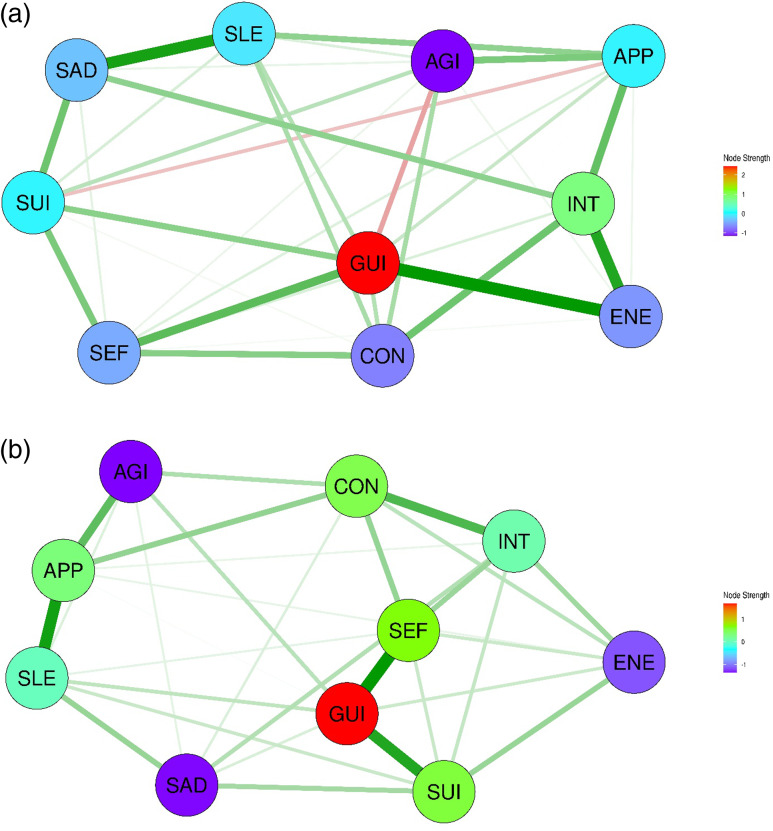
Network structures of 10 depressive symptoms in South or West Asian patients with depression. (a) Network structure of depressive symptoms in South or West Asian men with depression. (b) Network structure of depressive symptoms in South or West Asian women with depression. SAD, persistent sadness or low mood; INT, loss of interest or pleasure; ENE, fatigue or low energy; SLE, disturbed sleep; CON, poor concentration or indecisiveness; SEF, low self-confidence; APP, poor or increased appetite; SUI, suicidal thoughts or acts; AGI, agitation or slowing of movements; GUI, guilt or self-blame.

The baseline and clinical characteristics of the East Asia and South and West Asia subgroups are shown in Supplementary Tables 5 and 6. The networks for both sexes were composed of a single symptom cluster. As shown in Fig. 4(a) and 4(b), the network structure for the men in the South or West Asia subgroup showed high edge weights for ENE–GUI (0.376), SAD–SLE (0.346), and INT–ENE (0.327), whereas that for the women showed strong edges for SEF–GUI (0.346), SLE–APP (0.323), and SUI–GUI (0.303). GUI consistently exhibited the highest node strength centrality in the networks for both sexes, whereas AGI showed the lowest (Supplementary Figures 4a and 4b). The CS coefficients for the edge weights indicated an interpretable stability in both groups (CS coefficient: 0.284 for men, 0.361 for women), whereas all other centrality indices demonstrated very low stability. As shown in Table 3, the results of the NCT indicated no significant differences in either global strength or network structure invariance for the South or West Asia subgroup, regardless of whether the sample sizes were equalised or not. The Spearman correlation values for node centrality strength (*r* = 0.515) and edge weights (*r* = 0.801) revealed strong positive associations between the networks in the subgroup. Only one edge (2.2%) showed significant differences between the two groups.

## Discussion

In this study, we performed network analysis to explore the heterogeneity of depressive symptoms, defined according to NICE guidelines, among Asian men and women with depression. The results highlighted distinct differences in the network structures of depressive symptoms between sexes, emphasising the complex interplay of symptoms and their varying centralities.

### Sex-specific differences in depressive symptom networks

All 10 symptoms analysed in this study formed a single cluster in the networks for both men and women, indicating that the symptoms are highly interrelated and not separated into distinct groups regardless of sex. This highlights the challenge of administering therapeutic interventions to alleviate a specific symptom. The so-called *experimenter’s fat fingers problem* refers to difficulty in targeting a single symptom in isolation, as it is often interconnected with other symptoms, thereby unintentionally influencing them. In the current study, we observed that although the overall network structure of depression for both sexes appeared unified, the interconnections among symptoms were complex. Therefore, rather than attempting to establish clear causal relationships between symptoms, it is more important to precisely identify the symptoms likely to have greater downstream effects on others (Eronen, 2020).

In both men and women, APP, CON, GUI, INT, and SEF were identified as the top five nodes with the highest node strength centrality, whereas AGI, ENE, SAD, SLE, and SUI were the five nodes with the lowest node strength centrality. In men, SEF was the most central symptom, showing interconnections with GUI and CON. INT was identified as the most central node in women, showing significant edges with ENE, SAD, and CON. The high centrality of SEF and INT has been highlighted in previous studies. These studies indicated that SEF and INT are often closely interconnected with other symptoms, reinforcing their role in the maintenance of depression (Park *et al*., 2015; Park *et al*., 2021). In contrast, AGI showed the lowest node strength in men, whereas SLE exhibited the lowest node strength in women. This finding suggests that these symptoms may exert a ‘blinding’ effect on other symptoms. Psychomotor agitation or retardation is associated with poorer treatment outcomes and greater functional impairment. In addition, somatic symptoms such as sleep disturbances are frequently reported by women with depression (Fawcett, 1997; Geoffroy *et al*., 2025). However, the findings of the present study suggest that these symptoms play a relatively peripheral role within the overall depressive symptom network. This implies that pharmacological interventions targeting these symptoms may have a limited impact on the overall improvement of depressive disorders (Kuehner, 2003).

CS analysis of the depressive symptom network for men revealed that both betweenness and closeness centrality exhibited low stability, making their results less reliable. In contrast, the network for women demonstrated higher stability across all centrality indices, suggesting stronger structural integrity. The centrality of sadness (SAD) in women was distinct from that in men in this study. In women, SAD exhibited low node strength but demonstrated the second highest closeness and betweenness centrality after INT. This indicates that although SAD may not be directly connected to many other symptoms (low strength), it plays a crucial role in bridging connections between symptoms (high betweenness) and is more centrally positioned in the network (high closeness). In men, SAD was less prominent in both node strength and other centrality measures. This suggests that in women, sadness may act as a critical intermediary in the symptom network, influencing the overall connectivity and progression of depressive symptoms (Fried *et al*., 2016; Kim *et al*., 2023).

The NCT revealed a significant difference in global strength invariance between the networks for men and women (*p* = 0.020), suggesting that women exhibit a more densely interconnected network structure than men. However, when sample sizes were equalised across the groups, the difference in global strength was no longer significant (*p* = 0.446). This suggests that the observed global strength invariance may partially reflect statistical artefacts rather than structural differences. Moreover, the network structure invariance test did not yield statistically significant results (*p* = 0.535), indicating that the overall interconnections between symptoms were relatively similar between sexes. The results remained consistent even after equalising the sample sizes (*p* = 0.655), further supporting the theory that the general organisation of symptom networks between men and women is consistent.

Analysis of the entire study cohort, which was sufficiently large, yielded the most robust network stability. The network formed a single symptom cluster, and the overall structure and interrelations among symptoms closely resembled those observed in the network for women. This likely reflects the imbalanced distribution of men and women in the study population, which is a potential methodological limitation. However, the structural stability of the network for women was sufficiently preserved. INT exhibited the highest node strength, closeness, and betweenness centrality among all symptoms, demonstrating its prominent influence as a gateway symptom of depression. In contrast, although SAD, another gateway symptom, showed the second highest closeness and betweenness following INT, its node strength remained low, which is consistent with the clinical implications previously observed in women. Regarding edge weights, SEF–GUI, SLE–APP, INT–ENE, SAD–SUI, and SAD–INT displayed distinctly strong connections, each exceeding 0.25, which is consistent with findings from the symptom network for women.

### Implications of edge statistics

The edge statistics calculated in this study revealed several significant interconnections within the depressive symptom networks for both men and women. SEF–GUI, SLE–APP, SAD–SUI, INT–ENE, and SAD–INT had edge weights of 0.2 or higher in the networks for both sexes. These findings are consistent with those of previous studies that have highlighted the clustering of affective and neurovegetative symptoms within depressive symptom networks (Beck, 2008; Fried & Nesse, 2015). The strong interconnections between cognitive symptoms (e.g., SEF), affective symptoms (e.g., SAD and GUI), and high-risk symptoms (e.g., SUI) were particularly prominent. This underscores the necessity of administering interventions that target affective and cognitive symptoms of depression to alleviate high-risk symptoms.

The NCT results showed that the number of significantly different edges identified in the individual edge invariance test was 3 (6.7%). This indicates that most edge weights were not significantly different between men and women; however, differences in the overall patterns could still be observed. In the network for men, key interconnections such as SEF–GUI, SLE–APP, SAD–SUI, CON–SEF, INT–ENE, SAD–INT, and APP–AGI were identified as strong bonds. These connections form three distinct lines: ENE–INT–SAD–SUI, CON–SEF–GUI, and AGI–APP–SLE, representing clustering patterns around affective, cognitive, and neurovegetative symptoms, respectively. These patterns may suggest a more localised and distinct network structure, with clearer differentiation between symptom domains. The weak connectivity observed among symptom clusters suggests that each group of symptoms may exert pathological effects relatively independently. This implies that the overall treatment response may be diminished if the individual symptoms present in each patient are not accurately identified and managed. In the network for women, key interconnections such as SEF–GUI, INT–ENE, SLE–APP, SAD–INT, INT–CON, SAD–SUI, SUI–GUI, and CON–AGI were identified as strong bonds. The overall edge weights tended to be higher than those for men. Additionally, some edge weights in the network for women, such as INT–CON and ENE–SEF, were significantly higher than those for men. The strong interconnections among the symptoms suggest that pathological processes may be amplified through mutual reinforcement, potentially leading to the rapid worsening of symptoms. Accordingly, the importance of early intervention for the treatment of depression in women is particularly salient. Moreover, identifying and targeting central symptoms at an early stage may contribute significantly to overall symptom improvement. However, in cases where the condition has become chronic, the complex network of symptom interrelations may require multifaceted therapeutic approaches because relying solely on a one-dimensional, restricted approach often leads to subpar treatment efficacy (Filia *et al*., 2021).

### Regional differences in the network structures of depressive symptoms

The subgroup analysis conducted according to regional categories indicated that certain symptom pairs maintained high edge weights regardless of region or sex. For instance, SEF–GUI consistently showed a robust interconnection in every analysis, whereas SLE–APP, SAD–SUI, and INT–ENE exhibited significant associations in most analyses. This consistent high connectivity suggests that these symptom pairs likely share a strong neurobiological basis. Several neurobiological insights support these observations. Serotonin deficiency is linked to depressive mood, heightened aggression, impulsivity, and suicidal ideation (Wisłowska-Stanek *et al*., 2021). Dopamine deficiency, especially when associated with impaired reward circuitry, can lead to anhedonia and loss of motivational drive, resulting in fatigue (Treadway & Buckholtz, 2012). Moreover, previous research has indicated that typical neurovegetative symptoms can be distinguished from other symptoms based on specific genetic variants (Oliva *et al*., 2023).

Regional differences in the network structures of depressive symptoms were observed in this study. In Southeast Asia, INT emerged as a core symptom regardless of sex, with stronger connections such as SAD–INT and INT–APP. In South or West Asia, GUI was more central, with stronger edge weights in pairs such as ENE–GUI and SUI–GUI. Conversely, East Asia consistently demonstrated a statistically higher number of significant edges and higher average edge weights than other regions. In East Asia, both men and women displayed high interconnections between neurovegetative symptoms and other symptom domains. East Asian men exhibited the highest edge weight for APP–SLE (0.464), and at the threshold of significant edges (≥0.250), they showed the highest average edge weight overall. In contrast, East Asian women presented a more balanced pattern of strong interconnections among various symptoms.

The NCT further revealed a significant sex-specific difference in network structure exclusively within East Asia. Among men, only the East Asian subgroup showed two distinct symptom clusters: one comprising SAD, INT, SEF, SUI, and GUI, and the other comprising ENE, SLE, CON, APP, and AGI. These clusters correspond to the affective and neurovegetative domains, respectively, thereby highlighting the potential underpinning neurobiological factors. Recent studies have used data from genome-wide association studies to examine whether neurovegetative symptoms (SLE, APP, and ENE) share a common genetic basis (Goldman *et al*., 2010). Additionally, previous studies on genetics in East Asians have indicated that a high frequency of the BDNF Val66Met polymorphism and the 5-HTTLPR S-allele, which contribute to lower BDNF activity and reduced serotonin transporter expression, may elevate an individual’s predisposition to depression (Kendler *et al*., 2018; Tsai, 2018). These findings suggest that the abovementioned genetic vulnerability may have a specific influence on the manifestation of neurovegetative symptoms, as evidenced by the fact that the network structures for East Asians have higher edge weights for these symptoms than those for other regions. However, the significant sex-specific differences observed in the East Asian subgroup and the fact that only East Asian men showed two distinct symptom clusters suggest the presence of subtle neurobiological differences within. It is possible that either the expression levels of genes associated with neurovegetative symptoms are regulated differently by sex hormones or the more integrated neural network structure in women results in a single symptom cluster (Kang *et al*., 2020).

### Limitations

This study has certain limitations. First, the restriction of the range may have influenced the results, as all participants were diagnosed with depression, potentially reducing variance. This could have led to underestimating the relationship between symptom variability (standard deviation) and node strength centrality (Fried *et al*., 2016; Tsai, 2018). Additionally, although the NCT showed moderate similarity in node strength centrality between networks (Spearman’s correlation = 0.661), the restriction of the range may have introduced bias into the global strength invariance analysis. Second, the use of cross-sectional data limited the ability to differentiate between outdegree and indegree within the network, restricting the drawing of inferences regarding the directionality of symptom interactions (Bringmann *et al*., 2013). Third, potential confounding factors, such as age, symptom severity, or other clinical variables, were not controlled. This may have contributed to the sex-specific differences observed in the depressive symptom networks (Salokangas *et al*., 2002). Fourth, the possibility of sampling bias cannot be completely ruled out, as the data used were obtained from the REAP-AD study, which is not an epidemiological study. This suggests that the findings may not be fully generalisable to the broader population. Moreover, as all the participants were from Asian countries, the results of this study may not be applicable to global contexts. Fifth, the heterogeneity of the depressive symptoms may have influenced the observed network structures because symptom diversity and variability may have affected the correlations and interconnections between nodes. Sixth, as no standardised training was provided across participating sites to ensure uniform data collection and symptom assessment, variability in evaluation quality may have been introduced in the data collation process. Lastly, while the majority of CS coefficients for network centrality indices surpassed the recommended minimum threshold of 0.250, thus allowing for cautious interpretation, the coefficient for the depressive symptom network in Asian men was notably lower than 0.250. As a result, interpretations regarding this subgroup should be approached with greater caution. Despite this limitation, the overall trends observed may still yield valuable insights into the depressive symptomatology in this population.

### Conclusion

This study highlights sex-specific differences in the network structures of depressive symptoms among patients across various Asian countries. The findings of this study suggest that these differences may be driven more by specific symptom interconnections than the overall network structure. SEF and INT were identified as pivotal symptoms of depression in both men and women. However, the network for men was more localised, with SEF forming robust interconnections with various symptoms. The weak connectivity among symptom clusters indicates that each group may operate independently; consequently, if individual symptoms are not accurately identified and targeted, the overall treatment efficacy may be unsatisfactory. The symptom network for women was more diverse, with strong associations among symptoms, highlighting the prominent role of sadness. The strong interconnections among symptoms imply that mutual reinforcement can rapidly worsen the condition. This highlights the importance of early intervention targeting core symptoms and multifaceted therapeutic strategies for chronic cases. However, the subgroup analysis conducted according to regional classification revealed significantly distinct features in the East Asian population. East Asian men showed a clearer symptom grouping centred around neurovegetative symptoms, along with significant differences in overall network structures between sexes. Considering the findings of the previous studies on the neurobiological vulnerability of East Asians to depressive symptoms and the biological mechanisms underlying neurovegetative symptoms, the results of the current study suggest a need for further investigation focusing on depressive symptoms in East Asian populations.

## Supporting information

Kim et al. supplementary material 1 Kim et al. supplementary material

Kim et al. supplementary material 2 Kim et al. supplementary material

## Data Availability

The datasets generated or analysed during the study are not publicly available due to institutional ownership. However, they are available from the corresponding author upon reasonable request.
