# Supplementary material for: Sex differences in the network structures of depressive symptom profiles in Asian patients with depressive disorders: findings from the Research on Asian Psychotropic Patterns for Antidepressants, Phase 3
Source: Acta Neuropsychiatr. 2025 Jun 20;37:e70. doi: 10.1017/neu.2025.10020 (PMC13130287; doi:10.1017/neu.2025.10020)
Supplement: Kim et al. supplementary material 1 — Kim et al. supplementary material [file S0924270825100203sup001.docx]

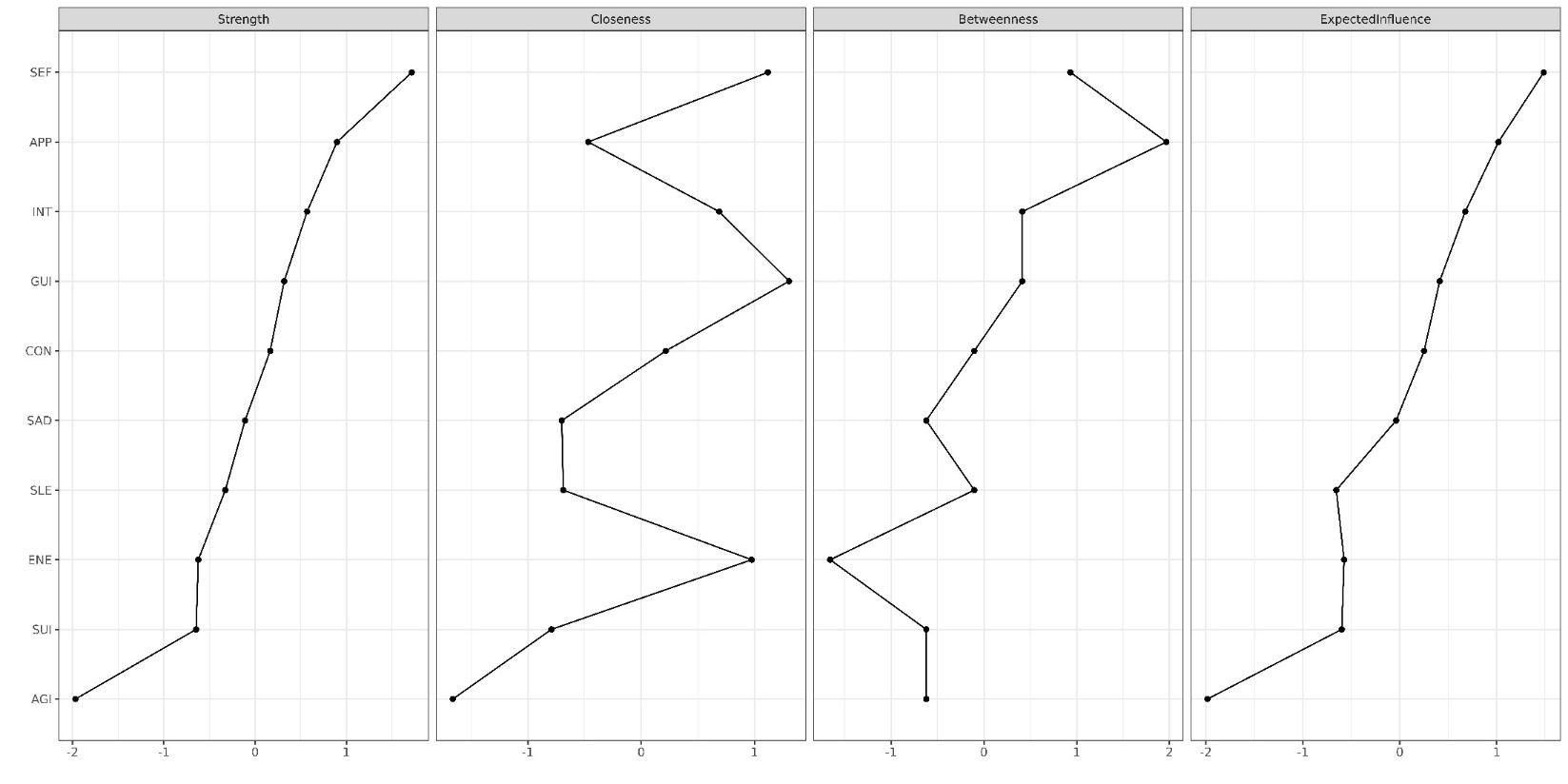


(a)


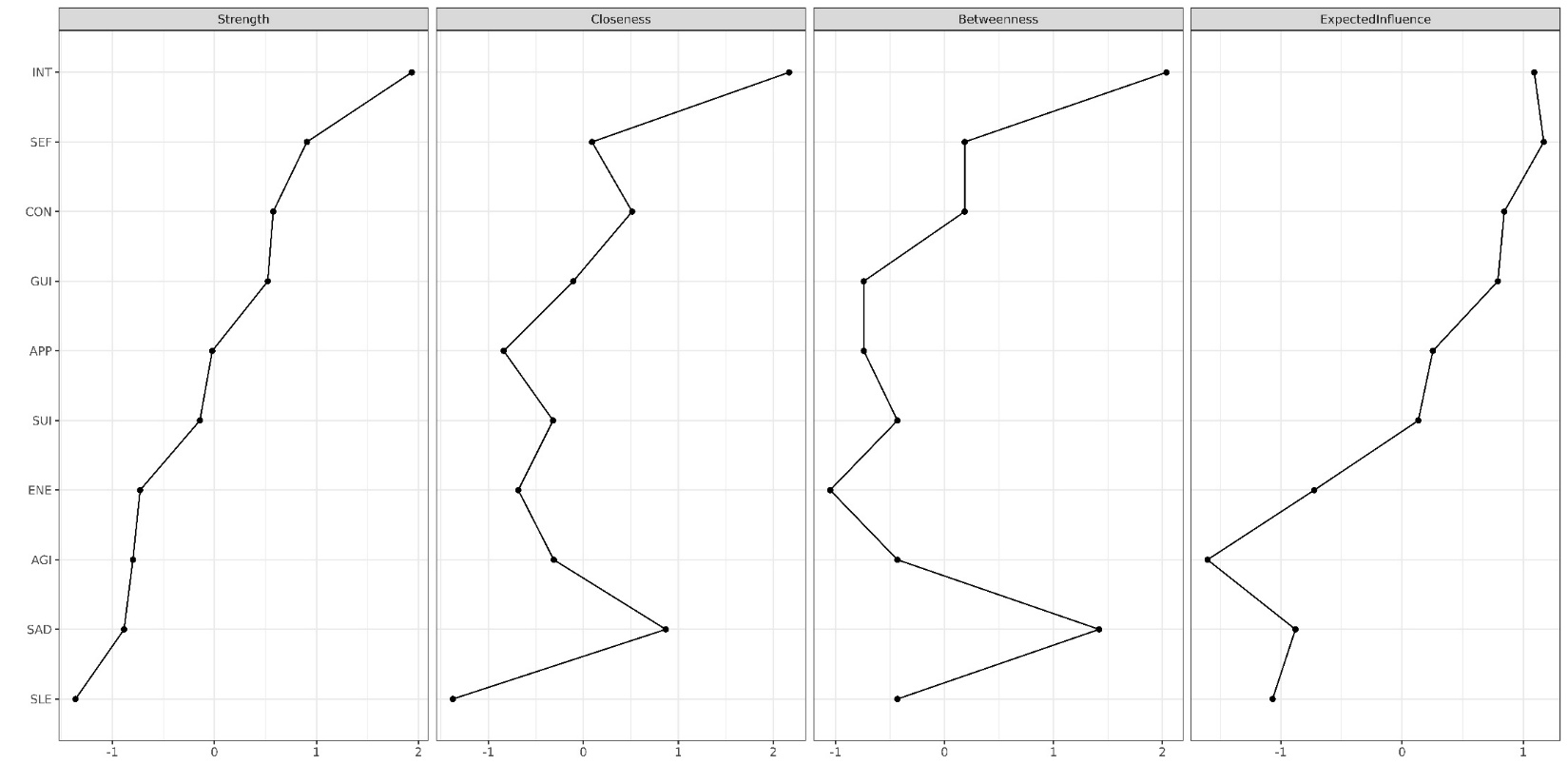


(b)


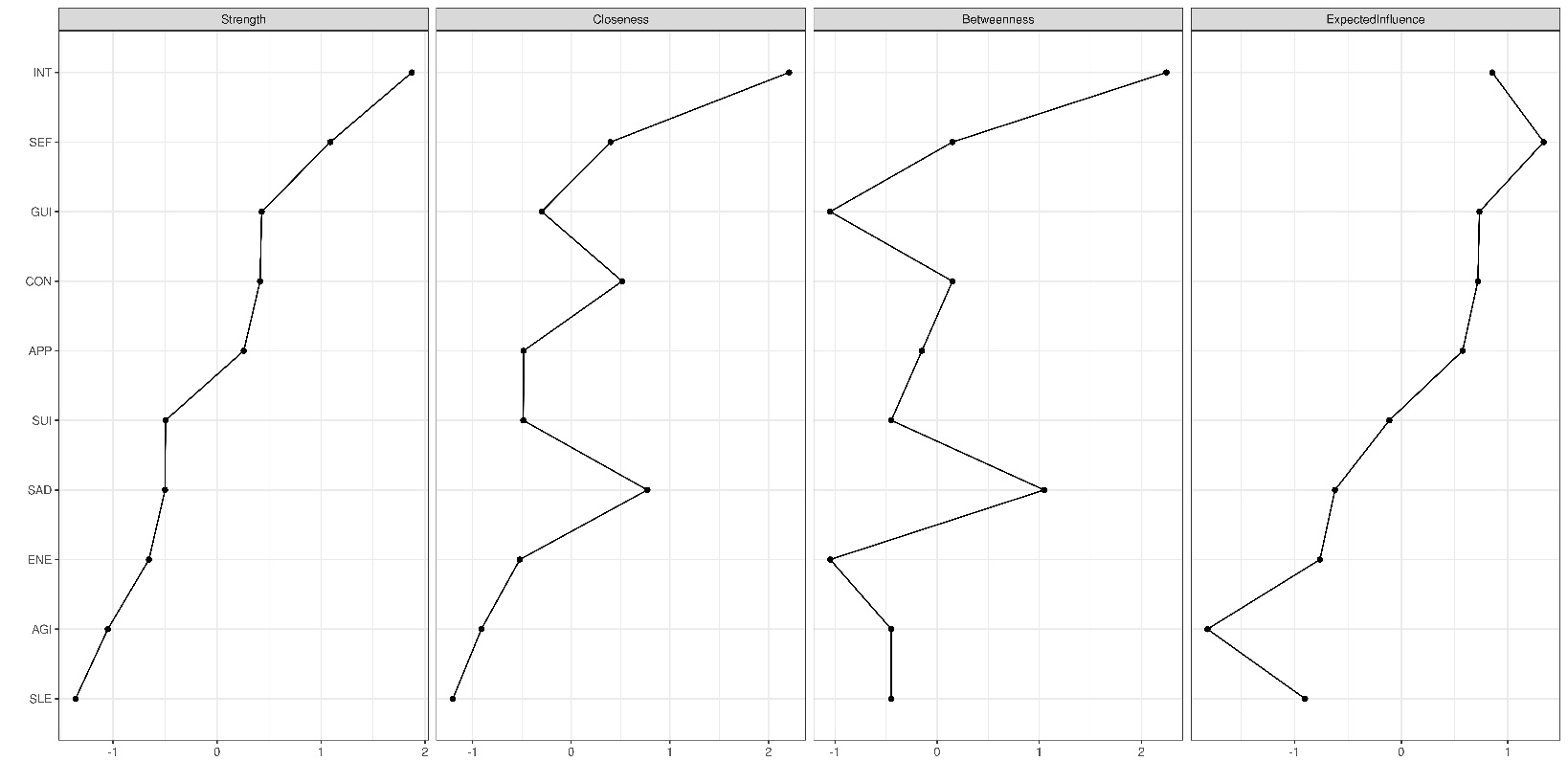


(c)

**Supplementary Figure 1.** Standardised node strength centrality, betweenness, and closeness of the estimated network structures for Asian patients with depression

(a) Standardised node strength centrality, betweenness, and closeness of the estimated network structures for Asian men with depression

(b) Standardised node strength centrality, betweenness, and closeness of the estimated network structures for Asian women with depression

(c) Standardised node strength centrality, betweenness, and closeness of the estimated network structures for Asian patients with depression

**SAD**, persistent sadness or low mood; **INT**, loss of interest or pleasure; **ENE**, fatigue or low energy; **SLE**, disturbed sleep; **CON**, poor concentration or indecisiveness; **SEF**, low self-confidence; **APP**, poor or increased appetite; **SUI**, suicidal thoughts or acts; **AGI**, agitation or slowing of movements; **GUI**, guilt or self-blame


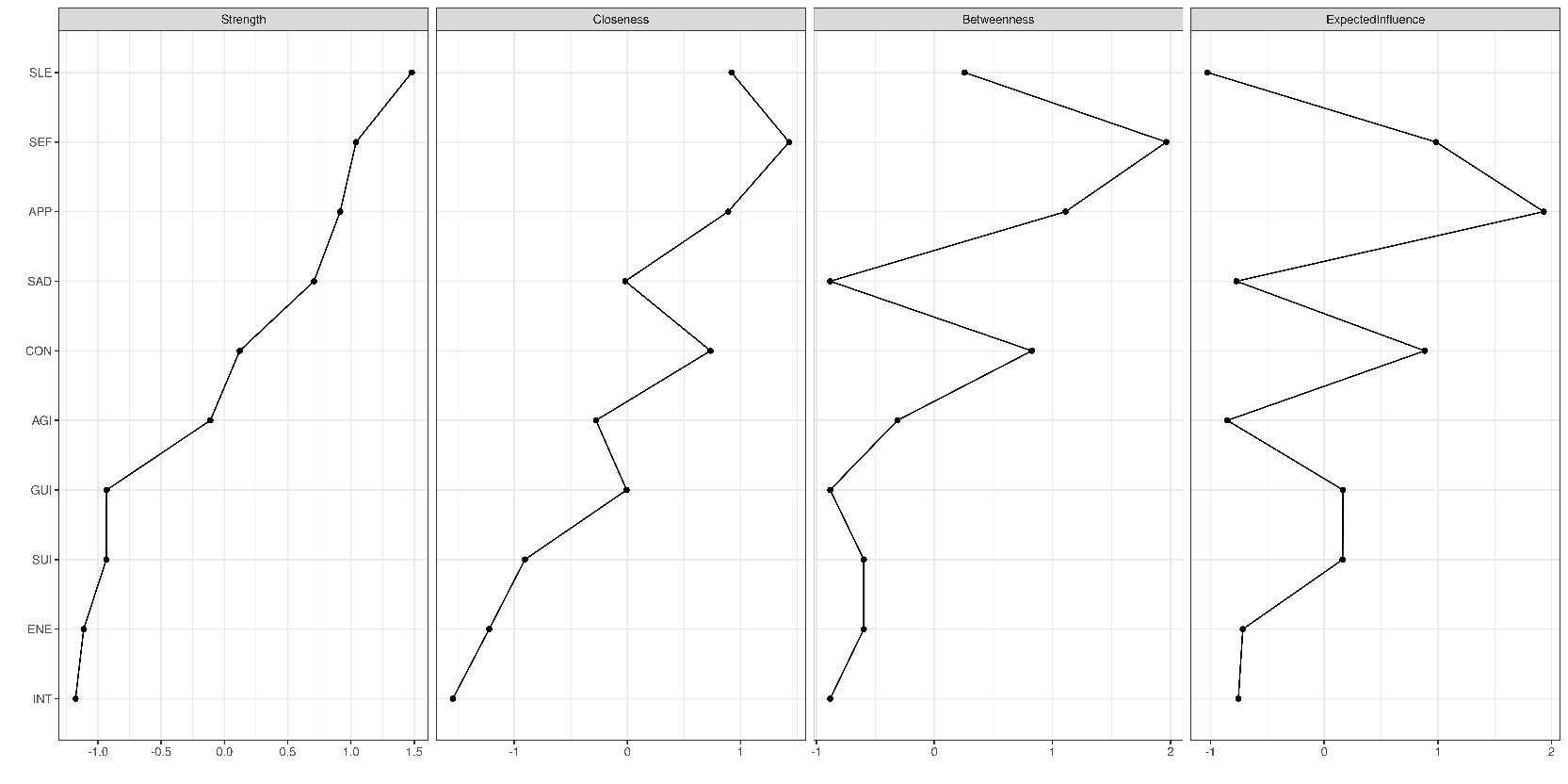


(a)


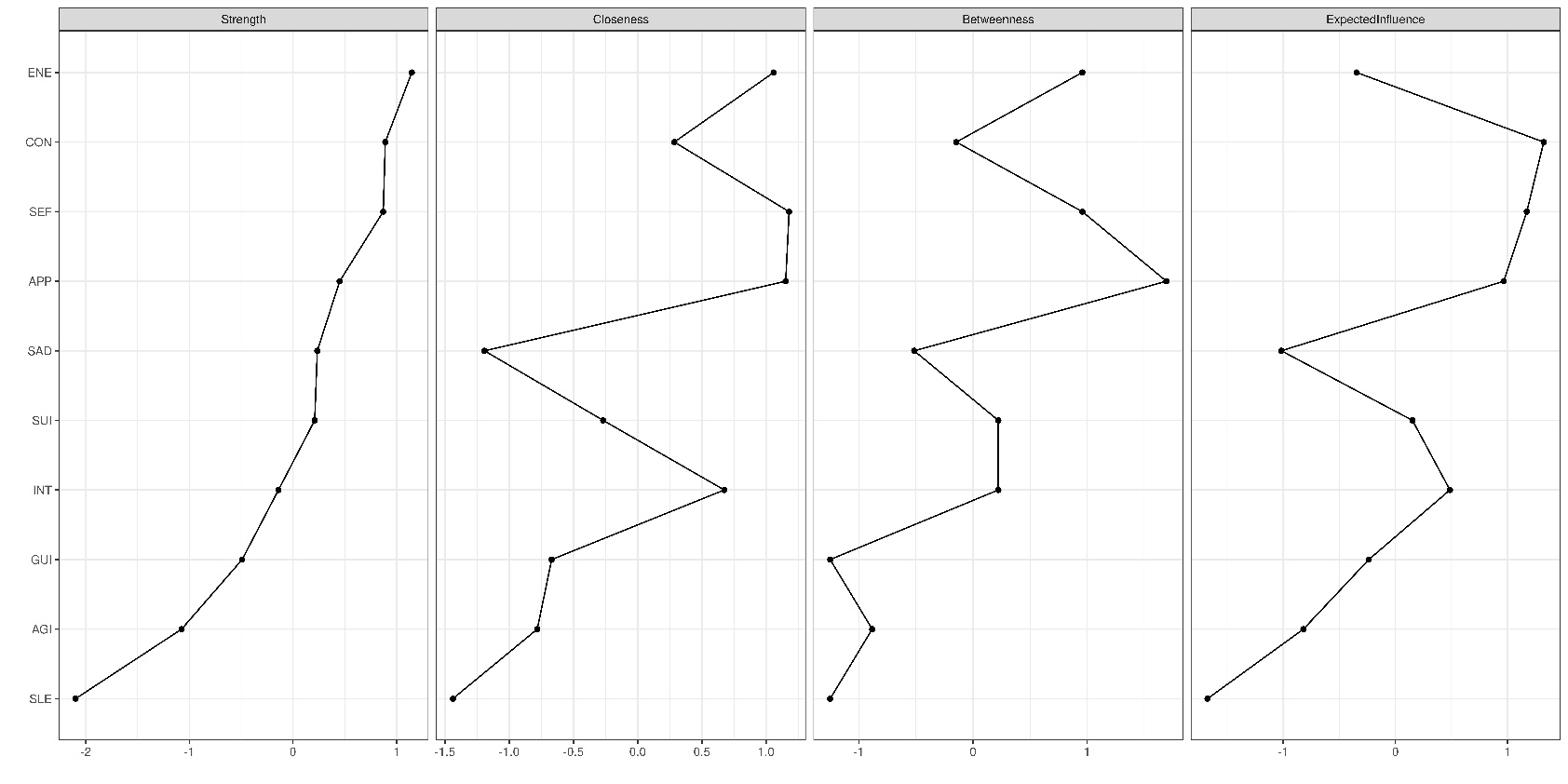


(b)

**Supplementary Figure 2.** Standardised node strength centrality, betweenness, and closeness of the estimated network structures for East Asian patients with depression

(a) Standardised node strength centrality, betweenness, and closeness of the estimated network structures for East Asian men with depression

(b) Standardised node strength centrality, betweenness, and closeness of the estimated network structures for East Asian women with depression

**SAD**, persistent sadness or low mood; **INT**, loss of interest or pleasure; **ENE**, fatigue or low energy; **SLE**, disturbed sleep; **CON**, poor concentration or indecisiveness; **SEF**, low self-confidence; **APP**, poor or increased appetite; **SUI**, suicidal thoughts or acts; **AGI**, agitation or slowing of movements; **GUI**, guilt or self-blame


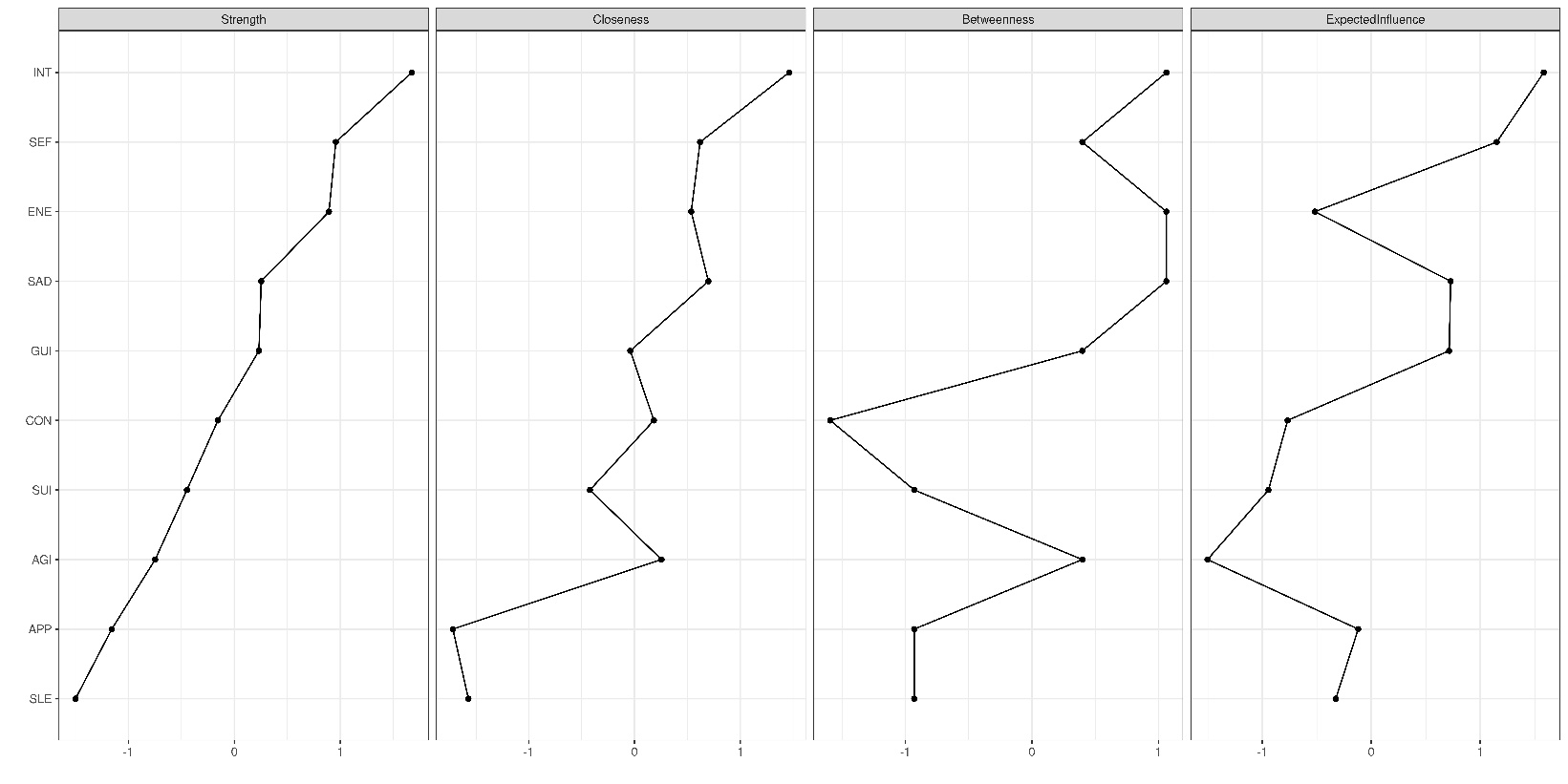


(a)


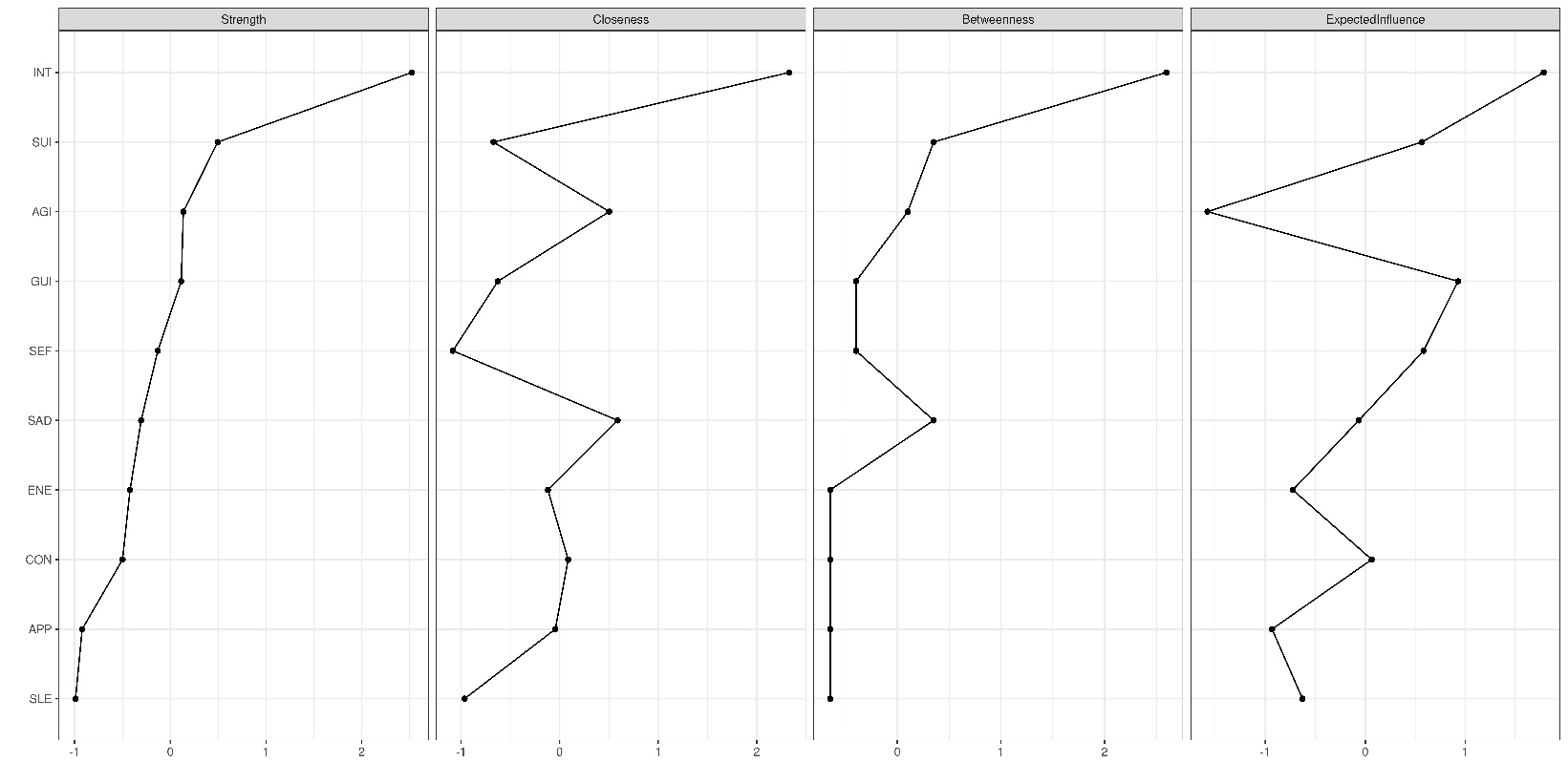


(b)

**Supplementary Figure 3.** Standardised node strength centrality, betweenness, and closeness of the estimated network structures for Southeast Asian patients with depression

(a) Standardised node strength centrality, betweenness, and closeness of the estimated network structures for Southeast Asian men with depression

(b) Standardised node strength centrality, betweenness, and closeness of the estimated network structures for Southeast Asian women with depression

**SAD**, persistent sadness or low mood; **INT**, loss of interest or pleasure; **ENE**, fatigue or low energy; **SLE**, disturbed sleep; **CON**, poor concentration or indecisiveness; **SEF**, low self-confidence; **APP**, poor or increased appetite; **SUI**, suicidal thoughts or acts; **AGI**, agitation or slowing of movements; **GUI**, guilt or self-blame


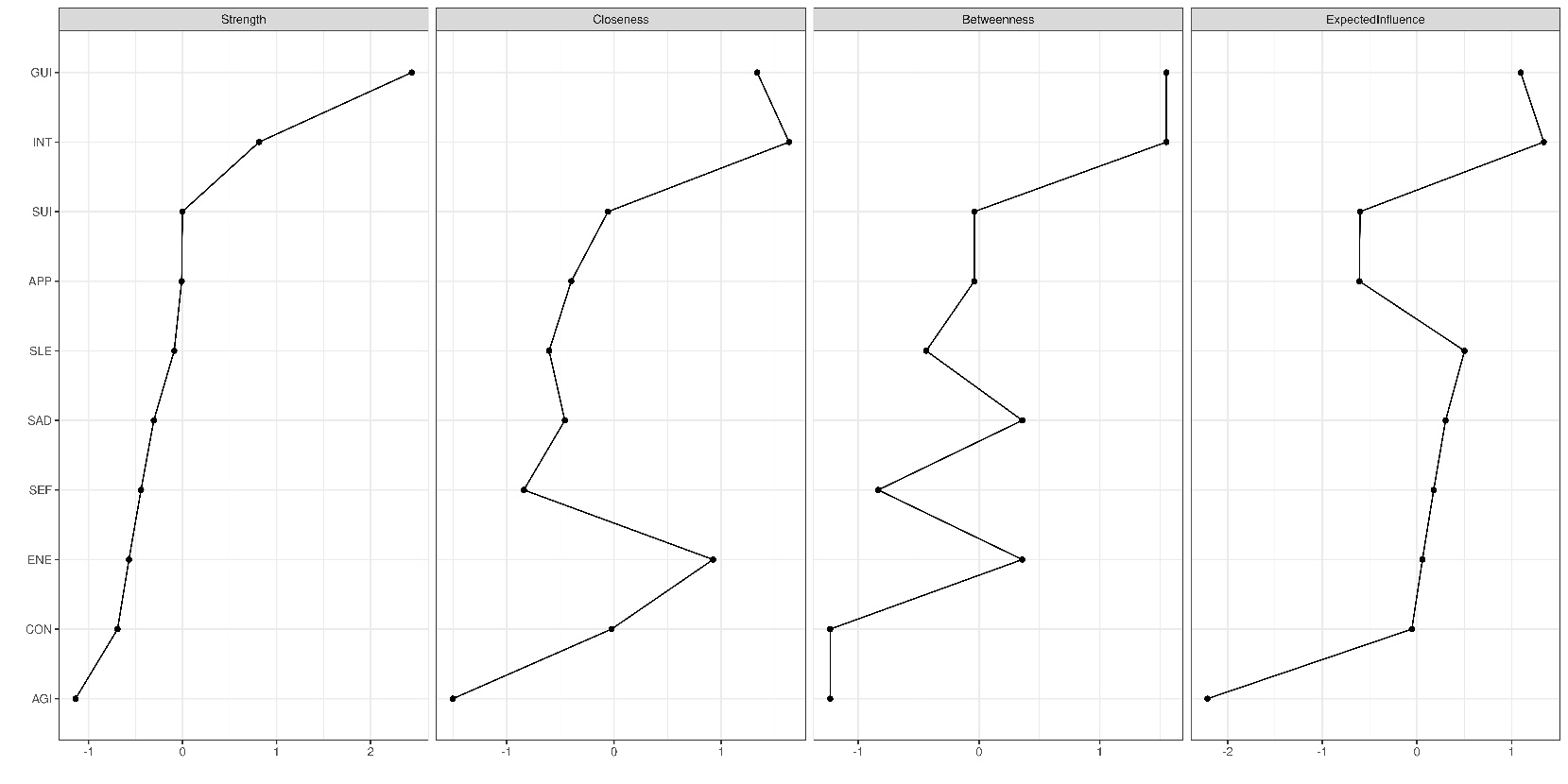


(a)


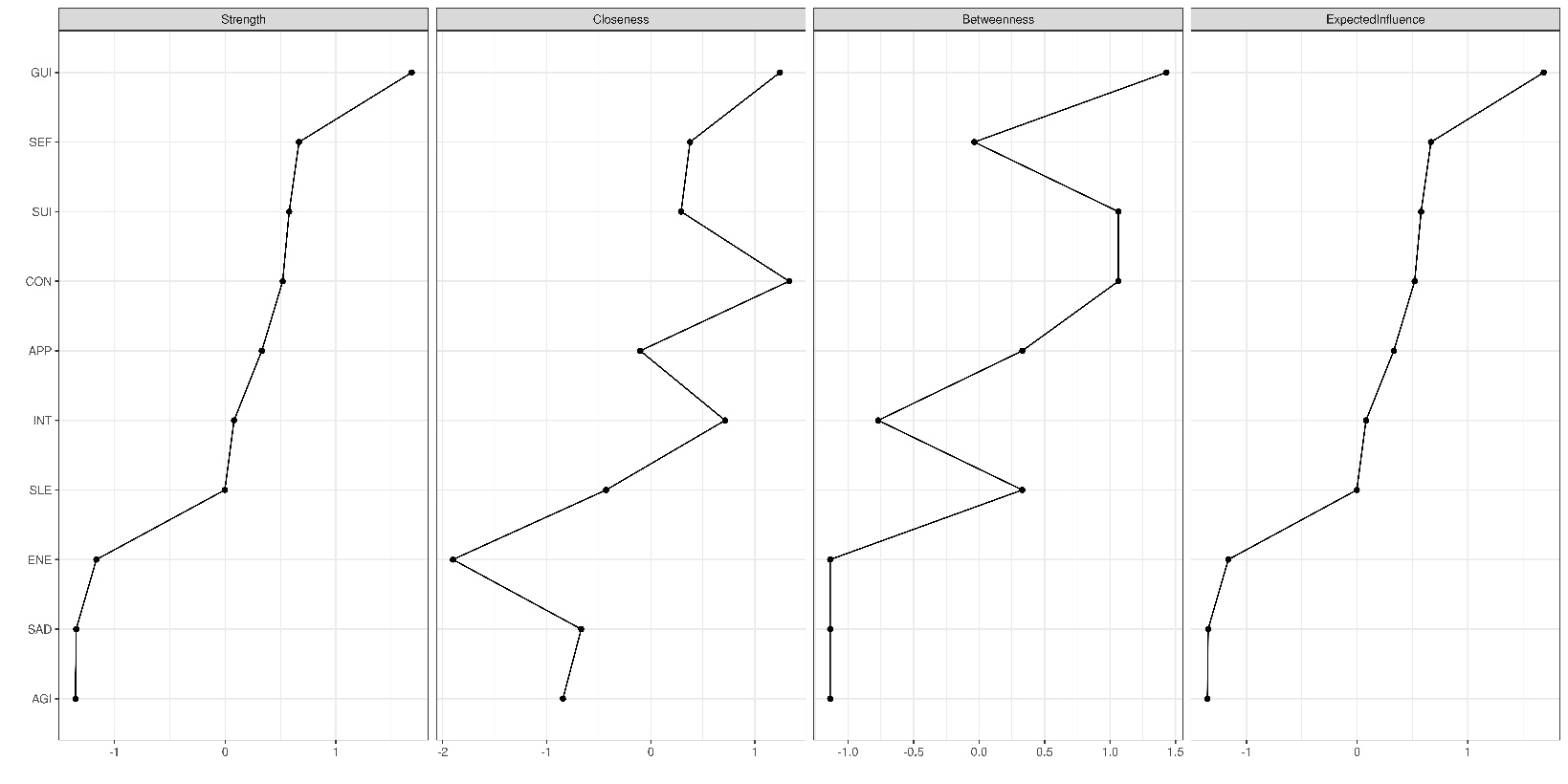


(b)

**Supplementary Figure 4.** Standardised node strength centrality, betweenness, and closeness of the estimated network structures for South or West Asian patients with depression

(a) Standardised node strength centrality, betweenness, and closeness of the estimated network structures for South or West Asian men with depression

(b) Standardised node strength centrality, betweenness, and closeness of the estimated network structures for South or West Asian women with depression

**SAD**, persistent sadness or low mood; **INT**, loss of interest or pleasure; **ENE**, fatigue or low energy; **SLE**, disturbed sleep; **CON**, poor concentration or indecisiveness; **SEF**, low self-confidence; **APP**, poor or increased appetite; **SUI**, suicidal thoughts or acts; **AGI**, agitation or slowing of movements; **GUI**, guilt or self-blame
