# Supplementary material for: Sex differences in the network structures of depressive symptom profiles in Asian patients with depressive disorders: findings from the Research on Asian Psychotropic Patterns for Antidepressants, Phase 3
Source: Acta Neuropsychiatr. 2025 Jun 20;37:e70. doi: 10.1017/neu.2025.10020 (PMC13130287; doi:10.1017/neu.2025.10020)
Supplement: Kim et al. supplementary material 2 — Kim et al. supplementary material [file S0924270825100203sup002.docx]

**Supplementary Table 1.** Baseline demographic and clinical characteristics of the participants in the East Asia subgroup

|  | **All participants**  **(n = 1,073)** | **Men**  **(n = 393)** | **Women (n = 680)** |
| --- | --- | --- | --- |
| Age, mean (SD) years | 41.1 (16.7) | 40.6 (16.3) | 41.4 (16.9) |
| Severity of depressive symptoms, n (%) |  |  |  |
| Subthreshold | 341 (31.8) | 136 (34.6) | 205 (30.1) |
| Mild | 185 (17.2) | 77 (19.6) | 108 (15.9) |
| Moderate | 260 (24.2) | 95 (24.2) | 165 (24.3) |
| Severe | 287 (26.7) | 85 (21.6) | 202 (29.7) |
| PHQ-9 score, n (%) |  |  |  |
| Minimal | 328 (30.6) | 110 (28.0) | 218 (32.1) |
| Mild | 222 (20.7) | 89 (22.6) | 133 (19.6) |
| Moderate | 372 (34.7) | 155 (39.4) | 217 (31.9) |
| Severe | 151 (14.1) | 39 (9.9) | 112 (16.5) |
| GAD-7 score, n (%) |  |  |  |
| Minimal | 461 (43.0) | 172 (43.8) | 289 (42.5) |
| Mild | 285 (26.6) | 119 (30.3) | 166 (24.4) |
| Moderate | 181 (16.9) | 65 (16.5) | 116 (17.1) |
| Severe | 146 (13.6) | 37 (9.4) | 109 (16.0) |
| Use of psychotropic drugs |  |  |  |
| Antipsychotic, n (%) | 359 (33.5) | 125 (31.8) | 234 (34.4) |
| Mood stabiliser, n (%) | 95 (8.9) | 47 (12.0) | 48 (7.1) |
| Anxiolytic, n (%) | 499 (46.5) | 177 (45.0) | 322 (47.4) |
| Hypnotic, n (%) | 173 (16.1) | 63 (16.0) | 110 (16.2) |

GAD-7, Generalised Anxiety Disorder-7; PHQ-9, Patient Health Questionnarie-9

**Supplementary Table 2.** Response frequency rates of the depressive symptom profiles of participants in the East Asia subgroup

|  | **All participants**  **(n = 1,073)** | **Men**  **(n = 393)** | **Women (n = 680)** |
| --- | --- | --- | --- |
| SAD, n (%) | 922 (85.9) | 328 (83.5) | 594 (87.4) |
| INT, n (%) | 716 (66.7) | 268 (68.2) | 448 (65.9) |
| ENE, n (%) | 666 (62.1) | 238 (60.6) | 428 (62.9) |
| SLE, n (%) | 746 (69.5) | 248 (63.1) | 498 (73.2) |
| CON, n (%) | 467 (43.5) | 159 (40.5) | 308 (45.3) |
| SEF, n (%) | 420 (39.1) | 147 (37.4) | 273 (40.1) |
| APP, n (%) | 368 (34.3) | 101 (25.7) | 267 (39.3) |
| SUI, n (%) | 334 (31.1) | 108 (27.5) | 226 (33.2) |
| AGI, n (%) | 292 (27.2) | 110 (28.0) | 182 (26.8) |
| GUI, n (%) | 374 (34.9) | 123 (31.3) | 251 (36.9) |

**SAD**, persistent sadness or low mood; **INT**, loss of interest or pleasure; **ENE**, fatigue or low energy; **SLE**, disturbed sleep; **CON**, poor concentration or indecisiveness; **SEF**, low self-confidence; **APP**, poor or increased appetite; **SUI**, suicidal thoughts or acts; **AGI**, agitation or slowing of movements; **GUI**, guilt or self-blame

**Supplementary Table 3.** Baseline demographic and clinical characteristics of the participants in the Southeast Asia subgroup

|  | **All participants**  **(n = 1,047)** | **Men**  **(n = 355)** | **Women (n = 692)** |
| --- | --- | --- | --- |
| Age, mean (SD) years | 38.5 (16.5) | 38.0 (15.6) | 38.8 (17.0) |
| Severity of depressive symptoms, n (%) |  |  |  |
| Subthreshold | 340 (32.5) | 115 (32.4) | 225 (32.5) |
| Mild | 129 (12.3) | 42 (11.8) | 87 (12.6) |
| Moderate | 275 (26.3) | 92 (25.9) | 183 (26.4) |
| Severe | 303 (28.9) | 106 (29.9) | 197 (28.5) |
| PHQ-9 score, n (%) |  |  |  |
| Minimal | 305 (29.1) | 108 (30.4) | 197 (28.5) |
| Mild | 189 (18.1) | 73 (20.6) | 116 (16.8) |
| Moderate | 410 (39.2) | 123 (34.6) | 287 (41.5) |
| Severe | 143 (13.7) | 51 (14.4) | 92 (13.3) |
| GAD-7 score, n (%) |  |  |  |
| Minimal | 400 (38.2) | 142 (40.0) | 258 (37.3) |
| Mild | 233 (22.3) | 83 (23.4) | 150 (21.7) |
| Moderate | 210 (20.1) | 69 (19.4) | 141 (20.4) |
| Severe | 204 (19.5) | 61 (17.2) | 143 (20.7) |
| Use of psychotropic drugs |  |  |  |
| Antipsychotic, n (%) | 253 (24.2) | 90 (36.1) | 163 (31.9) |
| Mood stabiliser, n (%) | 33 (3.2) | 10 (4.0) | 23 (4.5) |
| Anxiolytic, n (%) | 269 (25.7) | 93 (37.3) | 176 (34.4) |
| Hypnotic, n (%) | 184 (17.6) | 65 (26.1) | 119 (23.3) |

GAD-7, Generalised Anxiety Disorder-7; PHQ-9, Patient Health Questionnarie-9

**Supplementary Table 4.** Response frequency rates of the depressive symptom profiles of the participants in the Southeast Asia subgroup

|  | **All participants**  **(n = 1,047)** | **Men**  **(n = 355)** | **Women (n = 692)** |
| --- | --- | --- | --- |
| SAD, n (%) | 865 (82.6) | 286 (80.6) | 579 (83.7) |
| INT, n (%) | 701 (67.0) | 243 (68.5) | 458 (66.2) |
| ENE, n (%) | 618 (59.0) | 211 (59.4) | 407 (58.8) |
| SLE, n (%) | 658 (62.8) | 215 (60.6) | 443 (64.0) |
| CON, n (%) | 525 (50.1) | 201 (56.6) | 324 (46.8) |
| SEF, n (%) | 425 (40.6) | 148 (41.7) | 277 (40.0) |
| APP, n (%) | 376 (35.9) | 125 (35.2) | 251 (36.3) |
| SUI, n (%) | 366 (35.0) | 109 (30.7) | 257 (37.1) |
| AGI, n (%) | 277 (26.5) | 82 (23.1) | 195 (28.2) |
| GUI, n (%) | 273 (26.1) | 91 (25.6) | 182 (26.3) |

**SAD**, persistent sadness or low mood; **INT**, loss of interest or pleasure; **ENE**, fatigue or low energy; **SLE**, disturbed sleep; **CON**, poor concentration or indecisiveness; **SEF**, low self-confidence; **APP**, poor or increased appetite; **SUI**, suicidal thoughts or acts; **AGI**, agitation or slowing of movements; **GUI**, guilt or self-blame

**Supplementary Table 5.** Baseline demographic and clinical characteristics of the participants in the South or West Asia subgroup

|  | **All participants**  **(n = 793)** | **Men**  **(n = 250)** | **Women (n = 543)** |
| --- | --- | --- | --- |
| Age, mean (SD) years | 37.7 (16.6) | 37.2 (15.9) | 38.0 (16.9) |
| Severity of depressive symptoms, n (%) |  |  |  |
| Subthreshold | 188 (23.7) | 60 (24.0) | 128 (23.6) |
| Mild | 111 (14.0) | 36 (14.4) | 75 (13.8) |
| Moderate | 265 (33.4) | 69 (27.6) | 196 (36.1) |
| Severe | 229 (28.9) | 85 (34.0) | 144 (26.5) |
| PHQ-9 score, n (%) |  |  |  |
| Minimal | 230 (29.0) | 95 (38.0) | 135 (24.9) |
| Mild | 137 (17.3) | 43 (17.2) | 94 (17.3) |
| Moderate | 289 (36.4) | 76 (30.4) | 213 (39.2) |
| Severe | 137 (17.3) | 36 (14.4) | 101 (18.6) |
| GAD-7 score, n (%) |  |  |  |
| Minimal | 298 (37.6) | 113 (45.2) | 185 (34.1) |
| Mild | 179 (22.6) | 57 (22.8) | 122 (22.5) |
| Moderate | 167 (21.1) | 42 (16.8) | 125 (23.0) |
| Severe | 149 (18.8) | 38 (15.2) | 111 (20.4) |
| Use of psychotropic drugs |  |  |  |
| Antipsychotic, n (%) | 319 (40.2) | 120 (48.0) | 199 (36.6) |
| Mood stabiliser, n (%) | 54 (6.8) | 17 (6.8) | 37 (6.8) |
| Anxiolytic, n (%) | 325 (41.0) | 115 (46.0) | 210 (38.7) |
| Hypnotic, n (%) | 127 (16.0) | 40 (16.0) | 87 (16.0) |

GAD-7, Generalised Anxiety Disorder-7; PHQ-9, Patient Health Questionnarie-9

**Supplementary Table 6.** Response frequency rates of the depressive symptom profiles of the participants in the South and West Asia subgroup

|  | **All participants**  **(n = 793)** | **Men**  **(n = 250)** | **Women (n = 543)** |
| --- | --- | --- | --- |
| SAD, n (%) | 676 (85.2) | 206 (82.4) | 470 (86.6) |
| INT, n (%) | 586 (73.9) | 181 (72.4) | 405 (74.6) |
| ENE, n (%) | 475 (59.9) | 155 (62.0) | 320 (58.9) |
| SLE, n (%) | 542 (68.3) | 179 (71.6) | 363 (66.9) |
| CON, n (%) | 434 (54.7) | 143 (57.2) | 291 (53.6) |
| SEF, n (%) | 364 (45.9) | 121 (48.4) | 243 (44.8) |
| APP, n (%) | 353 (44.5) | 111 (44.4) | 242 (44.6) |
| SUI, n (%) | 278 (35.1) | 66 (26.4) | 212 (39.0) |
| AGI, n (%) | 247 (31.2) | 85 (34.0) | 162 (29.8) |
| GUI, n (%) | 226 (28.5) | 76 (30.4) | 150 (27.6) |

**SAD**, persistent sadness or low mood; **INT**, loss of interest or pleasure; **ENE**, fatigue or low energy; **SLE**, disturbed sleep; **CON**, poor concentration or indecisiveness; **SEF**, low self-confidence; **APP**, poor or increased appetite; **SUI**, suicidal thoughts or acts; **AGI**, agitation or slowing of movements; **GUI**, guilt or self-blame
